# Diagnostic accuracy of the WHO clinical staging system for detection of immunologically-defined advanced HIV disease: a systematic review and meta-analysis

**DOI:** 10.1111/hiv.70122

**Published:** 2025-09-26

**Authors:** Hussein H Twabi, Akitoshi Ueno, Josephine Ives, Ross Murtagh, Madalo Mukoka, Seyed Alireza Mortazavi, David S Lawrence, Augustine T Choko, Robina Semphere, Kelvin Balakasi, Ajay Rangaraj, Nathan Ford, Joseph N Jarvis, Peter MacPherson

**Affiliations:** 1 Public Health Group, Malawi-Liverpool-Wellcome Programme, Blantyre, Malawi; 2 Helse Nord TB Initiative, Kamuzu University of Health Sciences, Blantyre, Malawi; 3 Institute of Life Course and Medical Sciences, University of Liverpool, Liverpool, United Kingdom; 4 Clinical Research Department, London School of Hygiene and Tropical Medicine, London, United Kingdom; 5 Public Health Scotland, Glasgow, United Kingdom; 6 School of Infection and Immunity, University of Glasgow, Glasgow, United Kingdom; 7 Department of Infectious Disease Epidemiology and International Health, London School of Hygiene and Tropical Medicine, London, United Kingdom; 8 School of Health & Wellbeing, University of Glasgow, Glasgow, United Kingdom; 9 Botswana Harvard Health Partnership, Gaborone, Botswana; 10 School of Pathology, Faculty of Health Sciences, University of the Witwatersrand, Johannesburg, South Africa; 11 Department of Clinical Sciences, Liverpool School of Tropical Medicine, Liverpool, United Kingdom; 12 World Health Organization, Global HIV, Hepatitis and Sexually Transmitted Infections Programmes, Geneva, Switzerland; 13 Centre for Integrated Data and Epidemiological Research, University of Cape Town, South Africa

**Keywords:** HIV, clinical staging, screening, diagnostic accuracy, systematic review, meta-analysis.

## Abstract

**Introduction:**

People with advanced HIV disease (AHD) face high risks of severe illness and death. CD4 testing enables timely diagnosis and appropriate care, yet access remains limited in many settings. This review investigated the diagnostic accuracy of the World Health Organization (WHO) clinical staging for identifying AHD.

**Methods:**

We conducted a systematic review and meta-analysis of studies published between 1 January 1998 and 1 May 2024 that assessed both WHO clinical staging and CD4 counts in people living with HIV (PLHIV) aged five years and older (PROSPERO: CRD42024558372). We pooled sensitivity and specificity estimates of WHO Stage 3/4 for detecting AHD (CD4 <200 cells/μL) using bivariate random-effects meta-analysis. Risk of bias was assessed using QUADAS2, and certainty of evidence was appraised using GRADE.

**Results:**

Of 15,194 studies screened, 335 relevant studies were identified, from which 25 were included in evidence synthesis and 21 in the meta-analysis. Most studies were from the WHO African (19/25) and South-East Asian (5/25) regions. Risk of bias was moderate to high in 88% of studies, primarily due to issues with clinical staging assessment. Pooled sensitivity and specificity of WHO stage 3/4 were 60.7% (95% CI: 48.0–72.1%) and 72.4% (95% CI: 61.4–81.3%), respectively. Specificity was significantly higher outside the African region (p<0.001). In a population of 100,000 PLHIV with 30% AHD prevalence, WHO staging would miss 11,700 true AHD cases and misclassify 19,600.

**Conclusions:**

WHO clinical staging alone shows low accuracy for detecting AHD, risking both missed diagnoses and overtreatment. CD4 testing remains essential for accurately identifying and managing AHD.

## Introduction

An estimated 39 million people were living with HIV (PLHIV) in 2023, and 630,000 people died from HIV-related illnesses.[1] Advanced HIV disease (AHD), defined immunologically by the World Health Organization (WHO) as a CD4 count less than 200 cells per microlitre (μL),[2] affected approximately 4·3 million PLHIV in 2020,[3] increasing vulnerability to opportunistic infections, malignancies, and death.[4–6] The proportion of PLHIV with CD4 less than 200 cells/μL at antiretroviral therapy (ART) initiation has also been persistently high at around 30% globally since 2015.[7]

To address the challenge of AHD and reduce morbidity and mortality among PLHIV, WHO released guidelines for management of AHD in 2017.[2] Recommended interventions include screening, diagnosis, and treatment of opportunistic infections, chemoprophylaxis, ART initiation, and adherence and psychosocial support. Before appropriate HIV care and AHD interventions can be offered to PLHIV, health workers must be able to rapidly and accurately detect AHD, often in settings where laboratory access is limited.

CD4 cell count measurement is the recommended method for identifying AHD and monitoring treatment response in the absence of viral load measurement. However, access to CD4 testing has decreased since the introduction of the WHO “*Treat All”* strategy, whereby all PLHIV are eligible for ART regardless of CD4 cell count. WHO clinical staging has been used to define AHD, where a clinical stage of 3 or 4 in adults, adolescents, and children over five years of age is considered AHD – all under-five children are considered to have AHD at presentation. A recent global study found that between 2009 and 2019 the proportion of PLHIV initiating ART in southern Africa who had had a baseline CD4 count fell from 73% to 13%.[7] In an assessment of AHD diagnostic capacity of 32,946 health facilities from 15 African countries in 2024, only 58% had access to CD4 testing, with only 22% of eligible PLHIV receiving a CD4 test.[8] Additionally, due to decreasing demand, some CD4 cell count testing manufacturers have withdrawn from the market.[9, 10] Novel point-of-care tests for CD4 measurement have been developed but have suboptimal diagnostic accuracy in routine settings. [11, 12]

The WHO clinical staging system was developed out of a case definition for AIDS that was used for HIV testing and surveillance in an era when laboratory and point-of-care assays were not available.[13] WHO staging remains frequently used in clinical practice for identifying AHD in settings where CD4 testing is unavailable. Declining CD4 testing access means more individuals are either not assessed for AHD or are classified based on clinical staging alone.[13] A 2014 systematic review on the diagnostic accuracy of the WHO clinical staging system for ART eligibility among PLHIV in Africa found that against a CD4 cell count threshold of <200 cells/μL, the sensitivity of WHO stage 3/4 classification was 60% and the specificity was 73%.[14] Only a small number of studies had data available to contribute to this review. With the global scale up of ART and the increased significance of the 200 cells/µL threshold to identify AHD – due to the high risk of opportunistic illness and death once immunity declines below this level [15] – as an entry point to the AHD package of care, it is likely that more evidence is now available, and from a broader range of geographical settings.

As WHO staging is increasingly being used in place of CD4 measurement to identify AHD to inform the implementation of targeted interventions, it is critical to re-evaluate the accuracy of WHO staging in guiding AHD care in order to clarify the preferred approach for identifying AHD. To support the 2025 revision of the WHO guidelines for managing AHD, we updated and expanded the 2014 systematic review[14] to investigate the diagnostic accuracy of the WHO clinical staging system for identification of individuals with AHD as defined by a CD4 cell count of <200 cells/µL in PLHIV at any point of their individual history. We hypothesized that WHO clinical staging would have adequate sensitivity for detection of immunologically defined AHD to function as a clinical triage tool.

## Methods

We undertook a systematic review and meta-analysis which was registered in the international database of prospectively registered systematic reviews (PROSPERO: CRD42024558372).

### Inclusion and exclusion criteria

We included studies published between 1^st^ January 1998 and 1^st^ May 2024 that reported data with classification of PLHIV against WHO clinical staging system as the index test, and CD4 cell count as the reference standard. To align with WHO AHD guidelines, we only included studies from any country and published in any language where WHO clinical staging classification was compared at CD4 cell count threshold of <200 or ≤200 cells/μL.

We excluded case-control studies, conference abstracts, studies conducted among under-five children and without age disaggregated data to permit extraction of data for people aged five years or older, and studies that solely used lateral flow assays for classification of CD4 cell count. We did not include grey literature, unpublished studies, or incomplete studies found in trial registries.

### Search strategy and manuscript review

We searched six databases: Medline, Global Health, EMBASE, Global Index Medicus, Cochrane Library, and Africa-Wide Information. Search terms were determined after consultation with a librarian at the London School of Hygiene and Tropical Medicine, based on three domains: HIV/AIDS, WHO clinical staging and CD4 cell count. The full search strategy is shown in Supplementary Tables 1 and 2.

Following deduplication, titles and abstracts of all studies were reviewed against inclusion and exclusion criteria independently by pairs randomly allocated from a pool of 11 reviewers. Differences were resolved by discussion between reviewers and consensus with a third researcher.

Two randomly allocated reviewers independently reviewed the full text against inclusion and exclusion criteria and extracted data from eligible papers using a pre-tested Microsoft Form. Reasons for exclusion at full text review were recorded. We used the Rayyan web-based interface[16] to manage these processes and summarise the selection process in the Preferred Reporting Items for a Systematic Review and Meta-analysis (PRISMA) flow diagram.[17]

Aggregate data were extracted independently by two researchers and compared to ensure consistency. Discrepancies were resolved through discussion between reviewers and a third reviewer, and through comparison with study reports. Data extracted included: title; year of publication; digital object identifier (DOI); first author surname; country; WHO region; study design and period; study setting; participant recruitment approach; sample size; age; sex; index test information (WHO staging, including training provided, who conducted staging, and support tools provided); and reference test information (CD4 testing method, and classification threshold – either <200 cells/μL or ≤200 cells/μL). Data was extracted for the total number of participants, the number of people for each cell of a two-by-two table (true positive, false positive, true negative and false negative), sensitivity, specificity, positive predictive value (PPV) and negative predictive value (NPV) along with 95% confidence intervals, where reported.

*“True positive”* was defined as those with CD4 counts <200 cells/μL or ≤200 cells/μL who were correctly identified as stage 3 or 4 by WHO staging. *“False positive”* was defined as those with CD4 counts >200 cells/μL or ≥200 cells/μL who were incorrectly classified as stage 3 or 4 by WHO staging. *“False negative”* was defined as those with CD4 counts <200 cells/μL or ≤200 cells/μL who were not classified as stage 3 or 4 by WHO staging. *“True negative”* was defined as those with CD4 counts >200 cells/μL or ≥200 cells/μL who were correctly identified as not stage 3 or 4 by WHO staging.

### Statistical analysis

We described the characteristics of included studies, summarizing global regions and countries, study designs, recruitment settings, numbers of participants, age and sex profile. Our primary objective was to investigate the accuracy of WHO clinical stage 3 or 4 classification to identify immunologically confirmed AHD (CD4 <200 or ≤200 cells/μL). Using counts extracted into contingency tables from each study, we calculated the sensitivity and specificity for each study, along with binomial exact confidence intervals, and graphed these in forest plots, overall, and stratified by global region.

We constructed random-effects bivariate regression models for diagnostic test accuracy meta-analysis, jointly modelling logit-transformed sensitivity and logit-transformed specificity, assuming a bivariate normal distribution. The model was implemented using the *mada* (version 0.5.11) package in *R* statistical software (version 4.4.0).[18] From model estimates, we calculated the positive and negative likelihood ratios with 95% confidence intervals (CIs) estimated using the delta method. Using pooled sensitivity and specificity estimates, we projected the numbers of PLHIV who would be correctly and incorrectly classified as having AHD by WHO clinical staging in a hypothetical population of 100,000 people where the prevalence of AHD ranged from 0 to 100%.

*A priori*, we hypothesised that there were factors that would affect the performance of WHO clinical staging for AHD detection, including: cadre of health care worker performing the staging; whether the assessor of the staging was blinded to the patients’ CD4 cell count results; pre–training versus no prior training on WHO clinical staging; pre-*“Treat-all”* strategy versus post-*“Treat-all”* strategy (i.e. pre 2016 vs. post 2015); and global region (Africa vs others). We planned to investigate diagnostic accuracy stratified by these characteristics where sufficient data was available.

### Quality and risk of bias of selected studies

Quality and risk of bias was assessed using the Quality Assessment Tool for Diagnostic Accuracy Studies (QUADAS-2), which has signalling questions across four domains: patient selection, index test, reference standard, and flow and timing.[19] Applicability was assessed in patient selection, index test and reference standard domains. The results of risk of bias, shown as 'high risk', 'low risk' or 'unclear', were tabulated and summarised the evaluation of each domain in each study.

### GRADE assessment, within study variability and publication bias

The certainty of the evidence was assessed by the GRADE approach with five domains: risk of bias, indirectness, inconsistency, imprecision, and publication bias.[20] Following the approach described in the Cochrane Handbook for Systematic Reviews of Diagnostic Test Accuracy and prior WHO guideline evidence reviews,[21, 22] we assessed imprecision by examining the width of summary 95% CIs and downgraded to “imprecision” where the width was between 0·1 and 0·2, and “serious imprecision” where the width was >0·2, as these thresholds indicate a degree of uncertainty likely to influence clinical or policy decisions.[22] We evaluated inconsistency based on spread of point estimates of sensitivity and specificity from included studies, and by calculating *I*^*2*^ statistics. We assessed publication bias by inspecting funnel plots, and by expert judgement informed by knowledge of large multinational HIV studies.

## Results

Overall, 15,193 records were identified from database searching, with one additional paper identified by a subject matter expert. After deduplication, 8,208 unique records were reviewed and 7,873 were excluded at title and abstract screening. Of the 335 full text manuscripts reviewed, 310 were excluded, leaving 25 studies (totalling 52,866 participants) that met the study inclusion criteria.[23–47] Of these, 21 manuscripts, comprising 47,105 participants, contained data suitable for meta-analysis.[23–36, 40, 42–47] Reasons for exclusion at full text review are reported in Supplementary files. The commonest reason for exclusion was unavailability of data comparing WHO clinical staging against CD4 cell count.

The 25 included studies were conducted between 1994 and 2019, out of which 19 were conducted in the WHO African Region, with the remainder conducted in the Southeast Asian (n=5), or Eastern Mediterranean (n=1) Regions. The greatest number of studies were conducted in Uganda (n=5, including one multi-country study), Ethiopia (n=3), India (n=3), and Kenya (n=3, including one multi-country study). Studies were either prospectively recruited cross-sectional studies (n=19), or retrospective analyses of cross-sectional/cohort studies (n=6). Of the 25, 14 were additional studies that had not been included in the 2014 review.[23, 24, 27, 28, 30, 31, 33, 37, 38, 40–43, 46]

Participants were predominately recruited from outpatient clinics (n=20 studies). Sample sizes ranged from 103 to 19,525 participants (multi-country study). The proportion of males ranged from 0% to 77%, and the central age estimate (mean or median) ranged between 27 years and 57 years. Studies did not clearly report whether participants were ART naïve, or re-initiating ART.

Procedures for WHO staging were poorly reported, with 14/25 (56%) studies not specifying who performed the clinical staging, and the remainder (n=11) specifying this was medically qualified clinicians of various cadres. Only three studies reported that the person undertaking WHO clinical staging had received pretraining.[26, 39, 45] Two studies reported that the clinicians performing clinical staging had access to a checklist to support their classification;[28, 47] a separate study reported that the person performing clinical staging had weekly access to an expert infectious disease clinician.[35] Studies did not clearly report the microbiological, imaging or other specialist services available to support diagnosis of the AIDS-defining conditions. CD4 testing was primarily conducted using laboratory-based flow-cytometry measurement, with three studies reporting that measurement was done by the Coulter manual counting method.[36, 39, 46]

In the analysis of sensitivity of WHO stage 3/4 classification for detection of AHD, 22,869 participants contributed data. Study-level sensitivities ranged from 5·8% (565/9,687) to 89% (two studies: 76/85, and 263/297). The *I*^*2*^ statistic for heterogeneity was 99·6% (95% CI:99·6–99·7%). The pooled random-effects estimate for sensitivity was 60·7% (95% CI: 48·0–72·1%), with a 95% prediction interval of 10·9% to 95·1%.

In the analysis of specificity of WHO clinical stage 3/4 classification for detection of AHD, 24,236 participants contributed data. Study-level specificities ranged from 16·9% (10/59) to 96·4% (9,482/9,838). The *I*^*2*^ statistic for heterogeneity being 99·4% (95% CI: 99·3–99·5%). The pooled random-effects estimate for specificity was 72·4% (95% CI: 61·4– 81·3%), with a 95% prediction interval of 17·9% to 96·9%.

The positive likelihood ratio was 2·18 (0·28 to 17·16), and the negative likelihood ratio was 0·54 (0·11 to 2·56). Summary receiver operator characteristic curves from our bivariate random effects model can be seen in Supplementary Figure 1.

In subgroup analysis, the pooled sensitivity for studies conducted in the WHO African Region (62·0%, 95% CI: 47·0–74·9%) was slightly higher than for studies conducted in Southeast Asia and Eastern Mediterranean regions (54·6%, 95% CI: 36·3–71·7%), although this difference was not statistically significant (p=0·534). There was a statistically significant difference in specificity between studies conducted in the WHO African Region (67·3%, 95% CI: 54·2–78·2%) compared to the other regions (88·7%, 95% CI: 84·1– 92·1%); p<0·001. Further subgroup analysis by period before or after institution of WHO “*Treat all*” strategy – Supplementary figure 4 – showed a significantly (p=0·002) higher sensitivity in the pre-treat-all period (67·7%, 95% CI: 58·1–76·1%) as opposed to the post-treat-all period (20·2%, 95% CI: 6·8–46·9%), though there was no significant difference in specificities (p=0·236). There were insufficient data to permit subgroup analysis by other study characteristics.

In a hypothetical population of 100,000 PLHIV, where prevalence of AHD was 30%, 18,300 would be correctly classified as having AHD, 50,400 would correctly have AHD ruled out, 19,600 would be falsely classified as having AHD when they did not, and 11,700 would have AHD missed.

Nearly all studies had deficiency in reporting several items on risk of bias assessment, resulting in unclear judgement (Supplementary figure 2). Among items that were unclearly reported were whether WHO staging was conducted and interpreted independent of knowledge of CD4 cell count result, and whether there was an appropriately short time interval between performing WHO staging and measurement of CD4 cell count. Overall, 3/25 (12·0%) studies were judged to be at low risk of bias, 15/25 (60·0%) were judged to be at moderate risk of bias, and 7/25 (28.0%) were judged to be at high risk of bias. Funnel plots of both sensitivity and specificity indicated that there was likely to be publication bias (Supplementary Figure 3).

For assessment of both sensitivity and specificity, we judged that there were serious issues with risk of bias, indirectness, inconsistency, imprecision, and publication bias, hence there is low certainty in our overall summary estimates of accuracy.

## Discussion

This systematic review and meta-analysis, which included 52,866 participants from 25 studies from Africa, South-East Asia, and Eastern Mediterranean, showed that detection of AHD by WHO clinical staging had low accuracy. Pooled diagnostic accuracy estimates show that using WHO staging as a screening tool for AHD is likely to misclassify substantial numbers of PLHIV, resulting in missed opportunities to provide additional diagnostics, prophylaxis and treatments for opportunistic infections, or, otherwise, exposing individuals to unnecessary investigations and over-treatment.

We previously reviewed the evidence for accuracy of WHO clinical staging for detection of AHD in 2014, including studies conducted between 1994 and 2013.[14] In this updated review, we included 14 further studies in evidence synthesis. This review also included studies from all global regions, whereas the previous review focused only on Africa. Despite this widened search and larger amount of data included, our pooled estimates of sensitivity (this review: 61%, 95% CI: 48–72%; previous review: 60%,95% CI: 45–73%) and specificity (this review: 72%, 95% CI: 61–83%; previous review: 73%, 95% CI: 60–83%) were very similar. Only small gains in precision of pooled sensitivity and specificity were seen, likely due to the continued high degree of heterogeneity in included studies.

Although stratified estimates were heterogenous and imprecise, there was some evidence to suggest that specificity of WHO clinical staging for detection of AHD was higher in countries outside of the African Region. We speculate that this may be because access to clinical, laboratory, and imaging services may be greater in South-East Asia than Africa,[48] translating to a greater capacity to diagnose opportunistic infections and other conditions, increasing confidence in classification of PLHIV into WHO stage 3 and 4. Many conditions in WHO Stage 3 and 4 are challenging to diagnose without access to high-quality laboratory services, pathologists, and infectious disease specialists.[13, 49] It is notable that only two studies in the review clearly described which services were available to assist clinicians in diagnosing WHO stage 3 and 4 conditions.

We also found that diagnostic accuracy appeared to differ by study period, with sensitivity of WHO clinical staging being significantly higher in the pre-Treat All era (67·7%, 95% CI: 58·1–76·1%) compared with the post-Treat All era (20·2%, 95% CI: 6·8–46·9%), although there was no significant difference in specificity. Several explanations are possible. In the post–Treat All era, a much smaller fraction of PLHIV underwent CD4 testing at ART initiation, meaning that those who did receive CD4 measurement may have been systematically different from the broader patient population, thereby increasing the risk of bias. At the same time, declining use of CD4 testing may have reduced the clinical emphasis on actively diagnosing stage 3 and 4 conditions, contributing to lower sensitivity of clinical staging. Conversely, gradual improvements in laboratory and specialist diagnostic capacity in some settings may have enhanced the accuracy of excluding staging conditions, potentially contributing to higher specificity. Despite these observations, it is important to note that while stratification by study period is informative, the number of post-Treat All studies was limited, and potential biases in who received CD4 testing in recent years constrain interpretation. Nevertheless, these findings highlight the complex interplay between policy shifts, diagnostic availability, and staging performance, and underscore the limited reliability of clinical staging as a stand-alone tool for AHD detection in the current Treat All era.

In many high HIV prevalence countries, access to CD4 testing is extremely challenging.[7] Consequently, a large percentage of PLHIV are initiating (or resuming) ART without an accurate assessment for AHD.[8] The WHO clinical staging system may play a useful role in guiding individual clinical assessment and care, and has utility as a prognostic tool for future adverse outcomes.[50] However, its utility as a tool for identifying people with AHD – or indeed for population-based and programmatic surveillance of AHD – is limited, and possibly results in estimates that are biased by the setting in which it is conducted, and the resourcing of the supporting health system. Whilst alternative tools for detection of AHD have been investigated, including brief clinical assessments and biomarkers,[51, 52] none have shown sufficient accuracy. CD4 testing therefore remains the optimal approach both for individual clinical care, as well as for surveillance and programmatic monitoring. However, considerable effort and commitment from policymakers and funders will be required to scale-up availability of CD4 testing, particularly in low resource settings, and especially in Africa.[9] Additionally, although recent efforts have been unsuccessful,[11, 12] there is an urgent need for diagnostic developers to invest in accurate point-of-care tools for CD4 cell count measurement. Clear guidance from global policymakers signalling that CD4 measurement is the preferred tool for detection of AHD, and from national HIV departments indicating the utility of CD4 measurement for clinical care and surveillance, would increase market confidence.[10] Similar policy-driven market stimulation has successfully accelerated the global scale-up of other diagnostics, such as rapid point-of-care HIV testing and the GeneXpert MTB/RIF platform for tuberculosis, demonstrating that coordinated policy and procurement strategies can drive availability and uptake in resource-limited settings.

Despite the substantial body of evidence identified, our overall judgement was that there was very low confidence in the certainty of the estimates for sensitivity and specificity. Risk of bias was judged to be moderate to high for most (22/25, 88%) studies included, mainly related to the performance of WHO staging and measurement and interpretation of CD4 cell count results. Varying performance of WHO staging means that we are not confident that this index test was consistently applied across studies, likely explaining some of the large amount of heterogeneity identified. We also found that there was likely to be publication bias. It is challenging to design a search strategy that is sufficiently sensitive for identifying papers that might include comparisons of WHO staging with CD4 testing, and it is possible that some papers with relevant data were excluded. We identified no studies from the Americas, European, and Western Pacific regions, despite placing no restrictions on geography or language. Additionally, we are aware that in nearly all large national and multi-national HIV cohorts and registries, WHO staging and CD4 count are routinely recorded; yet we identified few studies arising from such studies. It is likely that there is a larger body of evidence available within HIV cohort databases, and that potentially the risk of bias in these may be lower. We also excluded four studies where sufficient data were not presented to permit inclusion in meta-analysis, although these exclusions are unlikely to have had a notable impact on our pooled estimates. All included studies were of adult populations, thus data for the accuracy of clinical staging systems in children are required. Studies did not clearly report whether participants were ART naïve, or re-initiating ART, and it is possible that the performance of WHO clinical staging differs between these groups.

## Conclusions

In conclusion, WHO clinical staging has low accuracy for detection of AHD. Our confidence in the accuracy estimates is very low due to high risk of bias, heterogeneity, imprecision, and publication bias. Where WHO clinical staging is used for individual clinical decisions, a substantial fraction of PLHIV is likely to be misclassified, resulting in missed opportunities for prophylaxis, diagnosis, and treatment of opportunistic conditions, as well as potential over-investigation and overtreatment, resulting in substantially increased downstream costs both to individuals and the health system. These limitations appear more pronounced in the absence of specific guidance or enhanced diagnostic support. Our findings suggest that, in its current form, WHO clinical staging alone is unlikely to be sufficient for reliably identifying AHD in many settings. We therefore recommend that national policymakers prioritize improving access to CD4 testing, and that diagnostic developers invest in more accurate and accessible technologies, to better support targeted AHD care and progress towards the United Nations Sustainable Development Goal of ending AIDS by 2030.[53]

## Supplementary Material

Supplementary materials

## Data Availability

Data collected for the study, including a data dictionary defining each field in the set, will be made available to others, along with related documents (study protocol, statistical analysis plan, analysis code) upon request from the corresponding author.
