# Supplementary material for: Diagnostic accuracy of the WHO clinical staging system for detection of immunologically-defined advanced HIV disease: a systematic review and meta-analysis
Source: HIV Med. Author manuscript; Available in PMC 2025 Oct 21. (PMC7618267; doi:10.1111/hiv.70122)
Supplement: Supplementary materials [file EMS209227-supplement-Supplementary_materials.docx]

Supplementary material

WHO Clinical Staging for Identification of AHD

[Supplementary Table 1. Search Strategy 2](#_Toc205881463)

[Supplementary Table 2. Studies excluded following full-text review 3](#_Toc205881464)

[Supplementary Figure 1. Summary receiver operator characteristic curve for WHO clinical stage 3 or 4 classification to detect advanced HIV disease 32](#_Toc205881465)

[Supplementary Figure 2. Risk of bias assessment 33](#_Toc205881466)

[Supplementary Figure 3. Funnel plots for sensitivity (A) and specificity (B) 34](#_Toc205881467)

[Supplementary figure 4. Forest plots for sensitivity and specificity of WHO clinical stage 3 or 4 classification for detection of advanced HIV disease stratified by period 35](#_Toc205881468)

[Supplementary figure 5. Forest plots for sensitivity and specificity of WHO clinical stage 3 or 4 classification for detection of advanced HIV disease stratified by period 36](#_Toc205881469)

[References 37](#_Toc205881470)

# Supplementary Table 1. Search Strategy

| **#** | **Search Terms** |
| --- | --- |
| 1 | HIV infections or HIV infection or HIV or hiv1 or hiv2 or hiv infec* or human immunodeficiency virus or human immunedeficiency virus or human immuno-deficiency virus or human immune-deficiency virus or (human immun* and deficiency virus) |
| 2 (MeSH) | exp hiv/ or exp hiv-1/ or exp hiv-2/ |
| 3 (MeSH) | exp HIV Infections/ |
| 4 | AIDS or acquired immunodeficiency syndrome or acquired immunedeficiency syndrome or acquired immuno-deficiency syndrome or acquired immune-deficiency syndrome or (acquired immun* and deficiency syndrome) |
| 5 (MeSH) | exp Acquired Immunodeficiency Syndrome/ |
| 6 | 1 or 2 or 3 or 4 or 5 |
| 7 | World Health Organisation clinical staging system or World Health Organization clinical stag* or WHO HIV AIDS clinical staging criteria or WHO HIV AIDS clinical stag* or WHO clinical staging system or WHO clinical staging for HIV or WHO clinical staging or WHO clinical stage or WHO clinical stage* or WHO stage or WHO staging or clinical stage or clinical staging or WHO stage condition or WHO staging condition or WHO condition* or (WHO stage and ("1" or "2" or "3" or "4" or I or II or III or IV)) or (WHO and ("1" or "2" or "3" or "4" or I or II or III or IV) and condition*) or (stage and ("1" or "2" or "3" or "4" or I or II or III or IV or "1/2" or "I/II" or "3/4" or "III/IV")) or AIDS defining illness* or AIDS-defining illness* or AIDS defining condition* or AIDS-defining condition* |
| 8 | CD4 count or CD4 counts or CD4 cell count or CD4 cell counts or CD4+ cell count or CD4+ cell counts or CD4 T-cell count or CD4 T-cell counts or lymphocyte count or lymphocyte counts or laboratory marker or laboratory markers |
| 9 (MeSH) | exp CD4 Lymphocyte Count/ |
| 10 | 8 or 9 |
| 11 | 6 and 7 and 10 |
| 12 | limit 11 to yr="1998 - 2024" |

MeSH = Medical Subject Headings

# Supplementary Table 2. Studies excluded following full-text review

| **#** | Author | Title | Year of publication | Reasons for excluding study |
| --- | --- | --- | --- | --- |
| 1 | Hilgartner et al. | Hematologic manifestations in HIV-infected children | 1991 | It is a review, case-control study or conference abstract |
| 2 | Armbruster et al. | Management of HIV-infected patients: To what extent are guidelines useful? - Results of a nationwide audit | 1998 | No data reported comparing CD4 vs. WHO stage |
| 3 | Junghans et al. | Uniform risk of clinical progression despite differences in utilization of highly active antiretroviral therapy: Swiss HIV Cohort Study | 1998 | No data reported comparing CD4 vs. WHO stage |
| 4 | Njobvu et al. | Spondyloarthropathy and human immunodeficiency virus infection in Zambia | 1998 | No data reported comparing CD4 vs. WHO stage |
| 5 | Yerly et al. | A Critical Assessment of the Prognostic Value of HIV-1 RNA Levels and CD4+ Cell Counts in HIV-Infected Patients | 1998 | No data reported comparing CD4 vs. WHO stage |
| 6 | Malamba et al. | The prognostic value of the World Health Organisation staging system for HIV infection and disease in rural Uganda | 1999 | No data reported comparing CD4 vs. WHO stage |
| 7 | Morgan et al. | HIV-1 RNA levels in an African population-based cohort and their relation to CD4 lymphocyte counts and World Health Organization clinical staging | 1999 | No data reported comparing CD4 vs. WHO stage |
| 8 | Wood et al. | Risk Factors for Developing Tuberculosis in HIV-1-Infected Adults From Communities With a Low or Very High Incidence of Tuberculosis | 2000 | No data reported comparing CD4 vs. WHO stage |
| 9 | Pezzotti et al. | Response to highly active antiretroviral therapy according to duration of HIV infection. | 2001 | No data reported comparing CD4 vs. WHO stage |
| 10 | Romero-Valdovinos et al. | [Quantification of T CD4+ lymphocytes and viral RNA in patients with HIV/AIDS]. | 2001 | No data reported comparing CD4 vs. WHO stage |
| 11 | Badri et al. | Effect of highly active antiretroviral therapy on incidence of tuberculosis in South Africa: A cohort study | 2002 | No data reported comparing CD4 vs. WHO stage |
| 12 | Barry et al. | The changing demographics of new HIV diagnoses at a London centre from 1994 to 2000 | 2002 | No data reported comparing CD4 vs. WHO stage |
| 13 | Damond et al. | Plasma RNA viral load in human immunodeficiency virus type 2 subtype A and subtype B infections. | 2002 | No data reported comparing CD4 vs. WHO stage |
| 14 | Egger et al. | Prognosis of HIV-1-infected patients starting highly active antiretroviral therapy: a collaborative analysis of prospective studies | 2002 | No data reported comparing CD4 vs. WHO stage |
| 15 | van der Loeff et al. | Mortality of HIV-1, HIV-2 and HIV-1/HIV-2 dually infected patients in a clinic-based cohort in The Gambia. | 2002 | No data reported comparing CD4 vs. WHO stage |
| 16 | Costello et al. | Predictors of low CD4 count in resource-limited settings: based on an antiretroviral-naive heterosexual Thai population. | 2005 | No data reported comparing CD4 vs. WHO stage |
| 17 | Lawn et al. | Early mortality among adults accessing a community-based antiretroviral service in South Africa: implications for programme design | 2005 | No data reported comparing CD4 vs. WHO stage |
| 18 | Lawn et al. | Tuberculosis among HIV-infected patients receiving HAART: long term incidence and risk factors in a South African cohort | 2005 | No data reported comparing CD4 vs. WHO stage |
| 19 | Mekonnen et al. | Low CD4 T cell counts before HIV-1 seroconversion do not affect disease progression in Ethiopian factory workers | 2005 | No data reported comparing CD4 vs. WHO stage |
| 20 | Obirikorang et al. | Predictors of low CD4 count in resource-limited settings: based on an antiretroviral-naive heterosexual Thai population | 2005 | No data reported comparing CD4 vs. WHO stage |
| 21 | Bekker et al. | Rapid scale-up of a community-based HIV treatment service: Programme performance over 3 consecutive years in Guguletu, South Africa | 2006 | No data reported comparing CD4 vs. WHO stage |
| 22 | Lynen et al. | The Added Value of a CD4 Count to Identify Patients Eligible for Highly Active Antiretroviral Therapy Among HIV-Positive Adults in Cambodia | 2006 | No data reported comparing CD4 vs. WHO stage |
| 23 | Nguyen et al. | Screening HIV-positive pregnant women for antiretroviral therapy: utility of self-reported symptoms | 2006 | No data reported comparing CD4 vs. WHO stage |
| 24 | Reuter et al. | Clinical staging of HIV/AIDS in infants and children | 2006 | No data reported comparing CD4 vs. WHO stage |
| 25 | Stringer et al. | Rapid scale-up of antiretroviral therapy at primary care sites in Zambia: Feasibility and early outcomes | 2006 | No data reported comparing CD4 vs. WHO stage |
| 26 | Sudha et al. | Western blot profile in HIV infection | 2006 | No data reported comparing CD4 vs. WHO stage |
| 27 | Zachariah et al. | Targeting CD4 testing to a clinical subgroup of patients could limit unnecessary CD4 measurements, premature antiretroviral treatment and costs in Thyolo District, Malawi | 2006 | No data reported comparing CD4 vs. WHO stage |
| 28 | Ajithkumar et al. | Impact of antiretroviral therapy on vocational rehabilitation | 2007 | No data reported comparing CD4 vs. WHO stage |
| 29 | Gimbel-Sherr et al. | Using nurses to identify HAART eligible patients in the Republic of Mozambique: results of a time series analysis | 2007 | No data reported comparing CD4 vs. WHO stage |
| 30 | Gitura et al. | Total lymphocyte count as a surrogate marker for CD4+ T cell count in initiating antiretroviral therapy at Kenyatta National Hospital, Nairobi | 2007 | No data reported comparing CD4 vs. WHO stage |
| 31 | Gupta et al. | Low sensitivity of total lymphocyte count as a surrogate marker to identify antepartum and postpartum Indian women who require antiretroviral therapy. | 2007 | No data reported comparing CD4 vs. WHO stage |
| 32 | MacLennan et al. | Diagnostic accuracy and clinical utility of a simplified low cost method of counting CD4 cells with flow cytometry in Malawi: diagnostic accuracy study | 2007 | Only includes children < 5years |
| 33 | Wadhwa et al. | AIDS-related opportunistic mycoses seen in a tertiary care hospital in North India. | 2007 | No data reported comparing CD4 vs. WHO stage |
| 34 | Bakeera-Kitaka et al. | Antiretroviral therapy for HIV-1 infected adolescents in Uganda: Assessing the impact on growth and sexual maturation | 2008 | No data reported comparing CD4 vs. WHO stage |
| 35 | Banda et al. | Antiretroviral therapy in the Malawi defence force: access, treatment outcomes and impact on mortality. | 2008 | No data reported comparing CD4 vs. WHO stage |
| 36 | Callens et al. | Computed CD4 percentage as a low-cost method for determining pediatric antiretroviral treatment eligibility | 2008 | No data reported comparing CD4 vs. WHO stage |
| 37 | Castilla et al. | Predictors of CD4+ cell count response and of adverse outcome among HIV-infected patients receiving highly active antiretroviral therapy in a public hospital in Peru | 2008 | No data reported comparing CD4 vs. WHO stage |
| 38 | Diro et al. | Assessment of risk behaviors and factors associated with oral and peri-oral lesions in adult HIV patients at Tikur Anbessa Specialized Hospital, Addis Ababa, Ethiopia | 2008 | No data reported comparing CD4 vs. WHO stage |
| 39 | Kiboneka et al. | Clinical and immunological outcomes of a national paediatric cohort receiving combination antiretroviral therapy in Uganda | 2008 | No data reported comparing CD4 vs. WHO stage |
| 40 | Makombe et al. | A national survey of prisoners on antiretroviral therapy in Malawi: access to treatment and outcomes on therapy. | 2008 | No data reported comparing CD4 vs. WHO stage |
| 41 | Makombe et al. | Antiretroviral Therapy in the Malawi Police Force: Access to Therapy and Treatment Outcomes | 2008 | No data reported comparing CD4 vs. WHO stage |
| 42 | Marazzi et al. | Excessive early mortality in the first year of treatment in HIV type 1-infected patients initiating antiretroviral therapy in resource-limited settings | 2008 | No data reported comparing CD4 vs. WHO stage |
| 43 | Bera et al. | Maternal outcomes following introduction of antiretroviral therapy in the public sector: A prospective study at a tertiary hospital in the Eastern Cape | 2009 | No data reported comparing CD4 vs. WHO stage |
| 44 | Brinkhof et al. | Mortality of HIV-infected patients starting antiretroviral therapy in sub-Saharan Africa: comparison with HIV-unrelated mortality. | 2009 | No data reported comparing CD4 vs. WHO stage |
| 45 | Collini et al. | Sustained Immunological Responses to Highly Active Antiretroviral Therapy at 36 Months in a Ghanaian HIV Cohort | 2009 | No data reported comparing CD4 vs. WHO stage |
| 46 | Johnson et al. | Total Lymphocyte Count and World Health Organization Pediatric Clinical Stage as Markers to Assess Need to Initiate Antiretroviral Therapy Among Human Immunodeficiency Virus-Infected Children in Moshi, Northern Tanzania | 2009 | No data reported comparing CD4 vs. WHO stage |
| 47 | Miiro et al. | Reduced morbidity and mortality in the first year after initiating highly active anti-retroviral therapy (HAART) among Ugandan adults | 2009 | No data reported comparing CD4 vs. WHO stage |
| 48 | Palombi et al. | Incidence and Predictors of Death, Retention, and Switch to Second-Line Regimens in Antiretroviral-Treated Patients in Sub-Saharan African Sites with Comprehensive Monitoring Availability | 2009 | No data reported comparing CD4 vs. WHO stage |
| 49 | Pathai et al. | Prevalence of HIV-associated ophthalmic disease among patients enrolling for antiretroviral treatment in India: a cross-sectional study | 2009 | No data reported comparing CD4 vs. WHO stage |
| 50 | Semitala et al. | Does toxicity to fixed dose stavudine, lamivudine and nevirapine regimen affect virologic suppression among HIV infected adults at the infectious diseases institute, Makerere university? | 2009 | It is a case-control study or conference abstract |
| 51 | Sivadasan et al. | High rates of regimen change due to drug toxicity among a cohort of South Indian adults with HIV infection initiated on generic, first-line antiretroviral treatment. | 2009 | No data reported comparing CD4 vs. WHO stage |
| 52 | Thejus et al. | The Functional status of patients with AIDS attending antiretroviral treatment center | 2009 | No data reported comparing CD4 vs. WHO stage |
| 53 | Tillekeratne et al. | Identifying HIV-infected children who may benefit from early initiation of antiretrovirals | 2009 | No data reported comparing CD4 vs. WHO stage |
| 54 | Tuboi et al. | Mortality during the first year of potent antiretroviral therapy in HIV-1-infected patients in 7 sites throughout latin America and the Caribbean | 2009 | No data reported comparing CD4 vs. WHO stage |
| 55 | van Oosterhout et al. | Diagnosis of antiretroviral therapy failure in Malawi: poor performance of clinical and immunological WHO criteria. | 2009 | No data reported comparing CD4 vs. WHO stage |
| 56 | Wools-Kaloustian et al. | The impact of the President's Emergency Plan for AIDS Relief on expansion of HIV care services for adult patients in western Kenya | 2009 | No data reported comparing CD4 vs. WHO stage |
| 57 | Agarwal et al. | High incidence of zidovudine induced anaemia in HIV infected patients in eastern India | 2010 | No data reported comparing CD4 vs. WHO stage |
| 58 | Athan et al. | Cost-effectiveness of routine and low-cost CD4 T-cell count compared with WHO clinical staging of HIV to guide initiation of antiretroviral therapy in resource-limited settings | 2010 | No data reported comparing CD4 vs. WHO stage |
| 59 | Boivin et al. | HIV-subtype A is associated with poorer neuropsychological performance compared to subtype D in ART-naïve Ugandan children | 2010 | No data reported comparing CD4 vs. WHO stage |
| 60 | Cesar et al. | Rates and reasons for early change of first HAART in HIV-1-infected patients in 7 sites throughout the Caribbean and Latin America. | 2010 | No data reported comparing CD4 vs. WHO stage |
| 61 | Charalambous et al. | Association of isoniazid preventive therapy with lower early mortality in individuals on antiretroviral therapy in a workplace programme | 2010 | No data reported comparing CD4 vs. WHO stage |
| 62 | Dembélé et al. | Incidence of tuberculosis after HAART initiation in a cohort of HIV-positive patients in Burkina Faso | 2010 | No data reported comparing CD4 vs. WHO stage |
| 63 | Descamps et al. | Increasing prevalence of transmitted drug resistance mutations and non-B subtype circulation in antiretroviral-naive chronically HIV-infected patients from 2001 to 2006/2007 in France | 2010 | No data reported comparing CD4 vs. WHO stage |
| 64 | Ekwom et al. | Prevalence and characteristics of articular manifestations in human imunodeficiency virus infection | 2010 | No data reported comparing CD4 vs. WHO stage |
| 65 | Emmett et al. | Predicting Virologic Failure Among HIV-1-Infected Children Receiving Antiretroviral Therapy in Tanzania: a Cross-Sectional Study | 2010 | No data reported comparing CD4 vs. WHO stage |
| 66 | Fawibe et al. | A retrospective evaluation of pretreatment and follow-up monitoring of HIV-infected adult patients in a resource-poor setting | 2010 | No data reported comparing CD4 vs. WHO stage |
| 67 | Gunawan et al. | The features of HIV-infected patients at A. Wahab Sjahranie General Hospital Samarinda, Indonesia | 2010 | It is a review, case-control study or conference abstract |
| 68 | Harries et al. | Baseline characteristics, response to and outcome of antiretroviral therapy among patients with HIV-1, HIV-2 and dual infection in Burkina Faso | 2010 | No data reported comparing CD4 vs. WHO stage |
| 69 | Hoffman et al. | Reducing mortality with co-trimoxazole preventive therapy at initiation of antiretroviral therapy in South Africa | 2010 | No data reported comparing CD4 vs. WHO stage |
| 70 | Janssen et al. | Successful paediatric HIV treatment in rural primary care in Africa | 2010 | No data reported comparing CD4 vs. WHO stage |
| 71 | Kouanfack et al. | WHO clinical criteria–based initiation of antiretroviral therapy: lessons from rural district hospitals in Cameroon with regard to 2009 revised WHO recommendations | 2010 | The paper does not use a CD4 threshold equal to or less than 200 |
| 72 | Kuhn et al. | Potential impact of new WHO criteria for antiretroviral treatment for prevention of mother-to-child HIV transmission | 2010 | The paper does not use a CD4 threshold equal to or less than 200 |
| 73 | Lee et al. | Profiling advanced disease in an Asian clinical human immunodeficiency virus cohort: comparison of two definitions for acquired immunodeficiency syndrome | 2010 | The paper does not use a CD4 threshold equal to or less than 200 |
| 74 | May et al. | Prognosis of HIV-1 infected patients starting antiretroviral therapy in sub-Saharan Africa: a collaborative analysis of scaleup programmes | 2010 | No data reported comparing CD4 vs. WHO stage |
| 75 | Mossdorf et al. | Impact of a national HIV voluntary counselling and testing (VCT) campaign on VCT in a rural hospital in Tanzania | 2010 | No data reported comparing CD4 vs. WHO stage |
| 76 | Mulissa et al. | Patients Present Earlier and Survival Has Improved, but Pre-ART Attrition Is High in a Six-Year HIV Cohort Data from Ethiopia | 2010 | No data reported comparing CD4 vs. WHO stage |
| 77 | Onifade et al. | Virologic and Immunologic Outcome of Treatment of HIV Infection with a Herbal Concoction, A-ZAM, Among Clients Seeking Herbal Remedy in Nigeria | 2010 | No data reported comparing CD4 vs. WHO stage |
| 78 | Tuboi et al. | Mortality Associated With Discordant Responses to Antiretroviral Therapy in Resource-Constrained Settings | 2010 | No data reported comparing CD4 vs. WHO stage |
| 79 | Baveewo et al. | Validation of World Health Organisation HIV/AIDS clinical staging in predicting initiation of antiretroviral therapy and clinical predictors of low CD4 cell count in Uganda | 2011 | Wrong CD4 cut off |
| 80 | Fatti et al. | Temporal trends in baseline characteristics and treatment outcomes of children starting antiretroviral treatment: An analysis in four provinces in South Africa, 2004-2009 | 2011 | No data reported comparing CD4 vs. WHO stage |
| 81 | Geng et al. | Trends in the clinical characteristics of HIV-infected patients initiating antiretroviral therapy in Kenya, Uganda and Tanzania between 2002 and 2009 | 2011 | No data reported comparing CD4 vs. WHO stage |
| 82 | Goel et al. | Lower genital tract infections in HIV-seropositive women in India | 2011 | No data reported comparing CD4 vs. WHO stage |
| 83 | Gomber et al. | Profile of HIV Infected Children from Delhi and Their Response to Antiretroviral Treatment | 2011 | Only includes children <5years, or it is not possible to disaggregate the rest of the study population from children <5 |
| 84 | Hamers et al. | HIV-1 drug resistance in antiretroviral-naive individuals in sub-Saharan Africa after rollout of antiretroviral therapy: a multicentre observational study | 2011 | No data reported comparing CD4 vs. WHO stage |
| 85 | Hoffmann et al. | Changing predictors of mortality over time from cART start: Implications for care | 2011 | No data reported comparing CD4 vs. WHO stage |
| 86 | Massimiliani et al. | HIV surveillance system and late presenters in Emilia-Romagna, 2006-2009 | 2011 | It is a review, case-control study or conference abstract |
| 87 | Matin et al. | Clinical Profile of HIV/AIDS-infected Patients Admitted to a New Specialist Unit in Dhaka, Bangladesh—A Low-prevalence Country for HIV | 2011 | No data reported comparing CD4 vs. WHO stage |
| 88 | Oudenhoven et al. | Total lymphocyte count is a good marker for HIV-related mortality and can be used as a tool for starting HIV treatment in a resource-limited setting | 2011 | No data reported comparing CD4 vs. WHO stage |
| 89 | Argemi et al. | Impact of malnutrition and social determinants on survival of HIV-infected adults starting antiretroviral therapy in resource-limited settings | 2012 | No data reported comparing CD4 vs. WHO stage |
| 90 | Arrive et al. | HIV Status Disclosure and Retention in Care in HIV Infected Adolescents on Antiretroviral Therapy (ART) in West Africa | 2012 | No data reported comparing CD4 vs. WHO stage |
| 91 | Cardoso et al. | Assessment of the Prognostic Value of the World Health Organization Clinical Staging System for HIV/AIDS in HIV-Infected Children and Adolescents in a Cohort in Belo Horizonte, Brazil | 2012 | No data reported comparing CD4 vs. WHO stage |
| 92 | Dash et al. | High incidence of zidovudine induced anaemia in HIV infected patients in southern odisha | 2012 | It is a case-control study or conference abstract |
| 93 | Deshmukh et al. | Clinico-epidemiological profile and quality of life of PLWHA in Central India | 2012 | It is a case-control study or conference abstract |
| 94 | Ferreyra et al. | Evaluation of clinical and immunological markers for predicting virological failure in a HIV/AIDS treatment cohort in Busia, Kenya | 2012 | No data reported comparing CD4 vs. WHO stage |
| 95 | Ganekal et al. | Evaluation of ocular manifestations and blindness in HIV/AIDS patients in a tertiary care hospital in south India. | 2012 | No data reported comparing CD4 vs. WHO stage |
| 96 | Kassa et al. | Incidence of tuberculosis and early mortality in a large cohort of HIV infected patients receiving antiretroviral therapy in a tertiary hospital in Addis Ababa, Ethiopia. | 2012 | No data reported comparing CD4 vs. WHO stage |
| 97 | Kayigamba et al. | Patient enrolment into HIV care and treatment within 90 days of HIV diagnosis in eight Rwandan health facilities: a review of facility-based registers. | 2012 | No data reported comparing CD4 vs. WHO stage |
| 98 | Kouanda et al. | Determinants and causes of mortality in HIV-infected patients receiving antiretroviral therapy in Burkina Faso: a five-year retrospective cohort study | 2012 | No data reported comparing CD4 vs. WHO stage |
| 99 | Maman et al. | Response to antiretroviral therapy: improved survival associated with CD4 above 500 cells/μ | 2012 | No data reported comparing CD4 vs. WHO stage |
| 100 | Patel et al. | Effect of antiretroviral therapy on clinical and immunologic disease progression in HIV positive children: One-year follow-up study. | 2012 | No data reported comparing CD4 vs. WHO stage |
| 101 | Sann Oo et al. | Clinical outcomes of patients on anti-retroviral therapy | 2012 | It is a case-control study or conference abstract |
| 102 | Tamí-Maury et al. | First report of HIV-related oral manifestations in Mali | 2012 | No data reported comparing CD4 vs. WHO stage |
| 103 | Zelalem et al. | Changes in nutritional, functional and immunological status of HIV-infected adults with antiretroviral therapy. | 2012 | No data reported comparing CD4 vs. WHO stage |
| 104 | Addis Alene et al. | Incidence and predictors of tuberculosis among adult people living with human immunodeficiency virus at the University of Gondar Referral Hospital, Northwest Ethiopia | 2013 | No data reported comparing CD4 vs. WHO stage |
| 105 | Bastard et al. | Adults receiving HIV care before the start of antiretroviral therapy in sub-Saharan Africa: patient outcomes and associated risk factors | 2013 | No data reported comparing CD4 vs. WHO stage |
| 106 | Chu et al. | Impact of tuberculosis on mortality among HIV-infected patients receiving antiretroviral therapy in Uganda: a prospective cohort analysis | 2013 | No data reported comparing CD4 vs. WHO stage |
| 107 | Damtie et al. | Common opportunistic infections and their CD4 cell correlates among HIV-infected patients attending at antiretroviral therapy clinic of Gondar University Hospital, Northwest Ethiopia | 2013 | No data reported comparing CD4 vs. WHO stage |
| 108 | De Beaudrap et al. | Morbidity after antiretroviral therapy initiation in HIV-1-infected children in West Africa: temporal trends and relation to CD4 count. | 2013 | No data reported comparing CD4 vs. WHO stage |
| 109 | Huibers et al. | Alternative markers to replace clinical staging in the decision to start anti-retroviral therapy (ART) in children when CD4 counts are not available | 2013 | It is a case-control study or conference abstract |
| 110 | Iroezindu et al. | Prevalence and risk factors for opportunistic infections in HIV patients receiving antiretroviral therapy in a resource-limited setting in Nigeria. | 2013 | No data reported comparing CD4 vs. WHO stage |
| 111 | Johnson et al. | Factors associated with timely initiation of antiretroviral therapy in two HIV clinics in Lilongwe, Malawi | 2013 | No data reported comparing CD4 vs. WHO stage |
| 112 | Kanters et al. | Increased mortality among HIV-positive men on antiretroviral therapy: survival differences between sexes explained by late initiation in Uganda | 2013 | No data reported comparing CD4 vs. WHO stage |
| 113 | Kone et al. | Epidemiological, clinical and therapeutics' data of HIV-infected patients placed on ART in the Segou hospital in Mali (2004-2011) | 2013 | No data reported comparing CD4 vs. WHO stage |
| 114 | Labhardt et al. | Outcomes of antiretroviral treatment programmes in rural Lesotho: health centres and hospitals compared | 2013 | No data reported comparing CD4 vs. WHO stage |
| 115 | Leroy et al. | Outcomes of antiretroviral therapy in children in Asia and Africa: A comparative analysis of the IeDEA pediatric multiregional collaboration | 2013 | No data reported comparing CD4 vs. WHO stage |
| 116 | Manjula et al. | Clinical and immunological features of pediatric HIV positive patients prior to anti-retroviral therapy-a cross sectional study | 2013 | Only includes children < 5years |
| 117 | Neupane et al. | Proportion of CD4 cells before and after antiretroviral therapy (ART) in people living with HIV/AIDS visiting ART center in Sukraraj Tropical and Infectious Disease Hospital, Teku, Kathmandu, Nepal | 2013 | No data reported comparing CD4 vs. WHO stage |
| 118 | Nguyen et al. | Outcomes of antiretroviral therapy in Vietnam: results from a national evaluation. | 2013 | No data reported comparing CD4 vs. WHO stage |
| 119 | Patten et al. | Impact on ART initiation of point-of-care CD4 testing at HIV diagnosis among HIV-positive youth in Khayelitsha, South Africa | 2013 | No data reported comparing CD4 vs. WHO stage |
| 120 | Rajeshwari et al. | Cardiac Abnormalities in HIV Infected Children Presenting to a Tertiary Level Teaching Hospital at New Delhi | 2013 | No data reported comparing CD4 vs. WHO stage |
| 121 | Ryavanki et al. | General profile and survival probabilities of HIV patients registered at Anti Retroviral Therapy (ART) Centre, New Civil Hospital, Surat, Gujarat | 2013 | No data reported comparing CD4 vs. WHO stage |
| 122 | Saka et al. | Loss of HIV-infected patients on potent antiretroviral therapy programs in Togo: risk factors and the fate of these patients | 2013 | No data reported comparing CD4 vs. WHO stage |
| 123 | Wei XiuQing et al. | Summary of anti-HIV treatment of AIDS patients in Hunan province of China during 2011. | 2013 | It is a review, case-control study or conference abstract |
| 124 | Agthe et al. | Lower anti-HIV-1 neutralization in HIV-infected children with CD4+ T cell depletion: opposite correlation to that in adults. | 2014 | No data reported comparing CD4 vs. WHO stage |
| 125 | Asmelash et al. | Predictors of suboptimal CD4 response among women achieving virologic suppression in a randomized antiretroviral treatment trial, Africa | 2014 | No data reported comparing CD4 vs. WHO stage |
| 126 | Bishnu et al. | Assessment of clinico-immunological profile of newly diagnosed HIV patients presenting to a teaching hospital of eastern India | 2014 | No data reported comparing CD4 vs. WHO stage |
| 127 | Costiniuk et al. | Lower baseline CD4 count is associated with a greater propensity to wards virological failure in a cohort of South African HIV patients | 2014 | It is a case-control study or conference abstract |
| 128 | Davies et al. | Prognosis of children with HIV-1 infection starting antiretroviral therapy in Southern Africa: A collaborative analysis of treatment programs | 2014 | Only includes children <5years, or it is not possible to disaggregate the rest of the study population from children <5 |
| 129 | Djarma et al. | Continuous free access to HAART could be one of the potential factors impacting on loss to follow-up in HAART-eligible patients living in a resource-limited setting: Ndjamena, Chad | 2014 | No data reported comparing CD4 vs. WHO stage |
| 130 | Eshetu et al. | Survival longevity of adult AIDS patients under ART: A case | 2014 | No data reported comparing CD4 vs. WHO stage |
| 131 | Fernández et al. | Long-term mortality and follow-up loss of patients on antiretroviral therapy | 2014 | No data reported comparing CD4 vs. WHO stage |
| 132 | Hao et al. | A retrospective cohort study on survival time of AIDS death cases receiving Antiretroviral Therapy and related factors | 2014 | No data reported comparing CD4 vs. WHO stage |
| 133 | Hoffmann et al. | Cotrimoxazole prophylaxis and tuberculosis risk among people living with HIV | 2014 | No data reported comparing CD4 vs. WHO stage |
| 134 | Kavya et al. | Cardiac manifestations in HIV | 2014 | WHO stage and CD4 count not performed on the same patient |
| 135 | Koech et al. | Characteristics and outcomes of HIV-infected youth and young adolescents enrolled in HIV care in Kenya | 2014 | No data reported comparing CD4 vs. WHO stage |
| 136 | Korir et al. | Developing Clinical Strength-of-Evidence Approach to Define HIV-Associated Malignancies for Cancer Registration in Kenya | 2014 | No data reported comparing CD4 vs. WHO stage |
| 137 | Lewden et al. | Disease patterns and causes of death of hospitalized HIV-positive adults in West Africa: a multicountry survey in the antiretroviral treatment era | 2014 | The paper does not use a CD4 threshold equal to or less than 200 |
| 138 | Lubis et al. | Anti-retroviral therapy of HIV infected patients | 2014 | It is a case-control study or conference abstract |
| 139 | Macpherson et al. | A novel community health worker tool outperforms WHO clinical staging for assessment of antiretroviral therapy eligibility in a resource-limited setting | 2014 | No data reported comparing CD4 vs. WHO stage |
| 140 | Molfino et al. | High attrition among HIV-infected patients with advanced disease treated in an intermediary referral center in Maputo, Mozambique | 2014 | No data reported comparing CD4 vs. WHO stage |
| 141 | Mugisha et al. | Determinants of mortality and loss to follow-up among adults Enrolled in HIV care services in Rwanda | 2014 | No data reported comparing CD4 vs. WHO stage |
| 142 | Mujugira et al. | Delay of antiretroviral therapy initiation is common in East African HIV-infected individuals in Serodiscordant partnerships | 2014 | No data reported comparing CD4 vs. WHO stage |
| 143 | Munthali et al. | Diagnostic accuracy of the WHO clinical staging system for defining eligibility for ART in sub-Saharan Africa: A systematic review and meta-analysis | 2014 | WHO stage and CD4 count not performed on the same patient |
| 144 | Nosyk et al. | Antiretroviral drug costs and prescription patterns in British Columbia, Canada 1996-2011 | 2014 | No data reported comparing CD4 vs. WHO stage |
| 145 | O'Hare et al. | Discordance between clinical and immunological ART eligibility criteria for children in Malawi | 2014 | No data reported comparing CD4 vs. WHO stage |
| 146 | Parvez et al. | Clinico-epidemiological profile of HIV positive patients attending ART centre at a tertiary care centre of north India | 2014 | No data reported comparing CD4 vs. WHO stage |
| 147 | Patten et al. | Advanced HIV disease at antiretroviral therapy (ART) initiation despite implementation of expanded ART eligibility guidelines during 2007-2012 in Khayelitsha, South Africa. | 2014 | It is a case-control study or conference abstract |
| 148 | Quartey  et al. | Comparison of retention and treatment outcomes between non-Muslims and Muslims in HIV Clinic Cohort | 2014 | It is a case-control study or conference abstract |
| 149 | Rao et al. | A retrospective cohort study of risk factors for death among human immunodeficiency virus infected adult patients | 2014 | It is a case-control study or conference abstract |
| 150 | Teasdale  et al. | CD4 cell decline and time to reaching ART eligibility in HIV patients from Rwanda | 2014 | It is a case-control study or conference abstract |
| 151 | Varma et al. | Characteristics of antiretroviral treatment-naïve patients with HIV-1 B and non-B subtypes in western Sydney | 2014 | It is a case-control study or conference abstract |
| 152 | Wen  et al. | Baseline Factors Associated with Mortality within Six Months after Admission among Hospitalized HIV-1 Patients in Shenyang, China | 2014 | No data reported comparing CD4 vs. WHO stage |
| 153 | Yirdaw et al. | Immunological recovery time of adult AIDS patients on ART: A case study at Felege-Hiwot Referral Hospital, Bahir-Dar,Ethiopia | 2014 | No data reported comparing CD4 vs. WHO stage |
| 154 | Zoufaly et al. | Determinants of HIV‐1 drug resistance in treatment‐naïve patients and its clinical implications in an antiretroviral treatment program in Cameroon | 2014 | It is a case-control study or conference abstract |
| 155 | Aliyu et al. | Enrolment trends in a comprehensive HIV programme in rural north-central Nigeria: improved care indices, but declining quality of clinical data over time | 2015 | No data reported comparing CD4 vs. WHO stage |
| 156 | Ayele et al. | Treatment outcomes and their determinants in HIV patients on anti-retroviral treatment program in selected health facilities of Kembata and Hadiya zones, southern nations, nationalities and peoples region, Ethiopia. | 2015 | No data reported comparing CD4 vs. WHO stage |
| 157 | Balestre et al. | CD4 response in treatment-naive HIV-2-infected patients: The IeDEA West Africa cohort | 2015 | It is a case-control study or conference abstract |
| 158 | Castelnuovo et al. | Long term virological outcomes of HIV infected patients on antiretroviral treatment in Uganda | 2015 | It is a case-control study or conference abstract |
| 159 | Castelnuovo et al. | Quantifying retention during pre-antiretroviral treatment in a large urban clinic in Uganda | 2015 | No data reported comparing CD4 vs. WHO stage |
| 160 | Dansereau et al. | Trends and determinants of antiretroviral therapy patient monitoring practices in Kenya and Uganda. | 2015 | No data reported comparing CD4 vs. WHO stage |
| 161 | Devi et al. | Implementation and operational research: high loss to follow-up among children on Pre-ART care under national AIDS program in Madurai, South India. | 2015 | No data reported comparing CD4 vs. WHO stage |
| 162 | Essomba et al. | Profil clinique et immunologique des patients infectes par le VIH a l'initiation du traitement antiretroviral a Douala. | 2015 | No data reported comparing CD4 vs. WHO stage |
| 163 | Ganga et al. | High Loss to Follow-up Among Children on Pre-ART Care Under National AIDS Program in Madurai South India | 2015 | No data reported comparing CD4 vs. WHO stage |
| 164 | Geng et al. | Estimation of mortality among HIV-infected people on antiretroviral treatment in east Africa: a sampling based approach in an observational, multisite, cohort study | 2015 | No data reported comparing CD4 vs. WHO stage |
| 165 | Haraka et al. | A bundle of services increased ascertainment of tuberculosis among HIV-infected individuals enrolled in a HIV cohort in rural sub-Saharan Africa | 2015 | No data reported comparing CD4 vs. WHO stage |
| 166 | Haskew et al. | Stage of HIV presentation at initial clinic visit following a community-based HIV testing campaign in rural Kenya. | 2015 | No data reported comparing CD4 vs. WHO stage |
| 167 | Hawkins et al. | High rates of early virologic failure in a cohort of Tanzanian HIV-infected adults | 2015 | It is a case-control study or conference abstract |
| 168 | Horumpende et al. | An Analysis of CLINICAL Signs and symptoms which best Predict the Need for Haart Initiation in HIV Infected South African Women | 2015 | It is a case-control study or conference abstract |
| 169 | Kamangu et al. | Virological profile of patients infected with HIV starting antiretroviral treatment in Kinshasa | 2015 | It is a case-control study or conference abstract |
| 170 | Katz et al. | Impact of South Africa's HIV treatment guidelines on early losses: A cohort analysis | 2015 | No data reported comparing CD4 vs. WHO stage |
| 171 | Kilama et al. | Patient level findings: Pre-ART mortality and its determinants in Tanzania public-driven HIV care program (2004-2011) | 2015 | It is a case-control study or conference abstract |
| 172 | Kondo et al. | Characterization of inpatient admission within a large HIV treatment program in Ethiopia | 2015 | No data reported comparing CD4 vs. WHO stage |
| 173 | Kumaraswamy et al. | A prospective study of highly active antiretroviral therapy in a tertiary-care hospital in south India | 2015 | No data reported comparing CD4 vs. WHO stage |
| 174 | Lee et al. | Improved retention in care for Ugandan youth living with HIV utilizing a youth-targeted clinic at entry to adult care: outcomes and implications for a transition model | 2015 | It is a case-control study or conference abstract |
| 175 | Liang et al. | Comparison of three staging systems in predicting prognosis for HIV/AIDS patients after initiating antiretroviral therapy | 2015 | No data reported comparing CD4 vs. WHO stage |
| 176 | Liping et al. | Quality of life of people living with HIV/AIDS: a cross-sectional study in Zhejiang province, China. | 2015 | No data reported comparing CD4 vs. WHO stage |
| 177 | Melaku et al. | Characteristics and outcomes of adult Ethiopian patients enrolled in HIV care and treatment: a multi-clinic observational study. | 2015 | No data reported comparing CD4 vs. WHO stage |
| 178 | Memish et al. | Antiretroviral therapy, CD4, viral load, and disease stage in HIV patients in Saudi Arabia: a 2001-2013 cross-sectional study | 2015 | WHO stage and CD4 count not performed on the same patient |
| 179 | Muhula et al. | Effects of highly active Anti-Retroviral Therapy on the survival of HIV-infected adult patients in urban slums of Kenya | 2015 | No data reported comparing CD4 vs. WHO stage |
| 180 | Nsanzimana et al. | Life expectancy among HIV-positive patients in Rwanda: a retrospective observational cohort study. | 2015 | No data reported comparing CD4 vs. WHO stage |
| 181 | Odeny et al. | The clock is ticking: the rate and timeliness of antiretroviral therapy initiation from the time of treatment eligibility in Kenya | 2015 | No data reported comparing CD4 vs. WHO stage |
| 182 | Teshome et al. | Do loss to follow-up and death rates from ART care vary across primary health care facilities and hospitals in south Ethiopia? A retrospective follow-up study | 2015 | No data reported comparing CD4 vs. WHO stage |
| 183 | Vanobberghe et al. | Challenges in estimating death and retention rates in a longitudinal cohort of HIV-infected persons in rural Tanzania | 2015 | WHO stage and CD4 count not performed on the same patient |
| 184 | Vinikoor et al. | Clinical correlates of alcohol use disorders among HIV-infected adults in Zambia | 2015 | It is a case-control study or conference abstract |
| 185 | Auld et al. | A Decade of Antiretroviral Therapy Scale-up in Mozambique: Evaluation of Outcome Trends and New Models of Service Delivery Among More Than 300,000 Patients Enrolled During 2004-2013 | 2016 | No data reported comparing CD4 vs. WHO stage |
| 186 | Balestre et al. | Immunologic response in treatment-naive HIV-2-infected patients: The IeDEA West Africa cohort | 2016 | No data reported comparing CD4 vs. WHO stage |
| 187 | Bastard et al. | Pediatric Access and Continuity of HIV Care Before the Start of Antiretroviral Therapy in Sub-Saharan Africa | 2016 | No data reported comparing CD4 vs. WHO stage |
| 188 | Bor et al. | Effect of eliminating CD4 thresholds on number of new art initiators in South Africa | 2016 | It is a case-control study or conference abstract |
| 189 | Herbas Ekat et al. | Is gender a factor associated with initiation of antiretroviral therapy at the Ambulatory Treatment Centre of Brazzaville? | 2016 | No data reported comparing CD4 vs. WHO stage |
| 190 | Kayigamba et al. | Discordant treatment responses to combination antiretroviral therapy in Rwanda: a prospective cohort study. | 2016 | No data reported comparing CD4 vs. WHO stage |
| 191 | Mammone et al. | How many people are living with undiagnosed HIV infection? An estimate for Italy, based on surveillance data | 2016 | WHO stage and CD4 count not performed on the same patient |
| 192 | McHugh et al. | Chronic Morbidity Among Older Children and Adolescents at Diagnosis of HIV Infection | 2016 | The paper does not use a CD4 threshold equal to or less than 200 |
| 193 | Monika et al. | Evaluating appointment patterns to improve sustainability of HIV treatment in Zambia | 2016 | It is a review, case-control study or conference abstract |
| 194 | Nlend et al. | Predictors of mortality among HIV-infected children receiving highly active antiretroviral therapy | 2016 | No data reported comparing CD4 vs. WHO stage |
| 195 | O'Brien et al. | Risk factors for mortality during antiretroviral therapy in older populations in resource-limited settings | 2016 | WHO stage and CD4 count not performed on the same patient |
| 196 | Oo et al. | Alarming attrition rates among HIV-infected individuals in pre-antiretroviral therapy care in Myanmar, 2011–2014 | 2016 | No data reported comparing CD4 vs. WHO stage |
| 197 | Sasse et al. | Late presentation to HIV testing is overestimated when based on the consensus definition. | 2016 | No data reported comparing CD4 vs. WHO stage |
| 198 | Schooley et al. | CD4 Variability in Malawi: Implications for Use of a CD4 Threshold of 500 Cells/mm3 Versus Universal Eligibility for Antiretroviral Therapy | 2016 | No data reported comparing CD4 vs. WHO stage |
| 199 | Seyoum et al. | Quasi-Poisson versus negative binomial regression models in identifying factors affecting initial CD4 cell count change due to antiretroviral therapy administered to HIV-positive adults in North-West Ethiopia (Amhara region). | 2016 | No data reported comparing CD4 vs. WHO stage |
| 200 | Takah et al. | Delayed entry into HIV care after diagnosis in two specialized care and treatment centres in Cameroon: the influence of CD4 count and WHO staging | 2016 | No data reported comparing CD4 vs. WHO stage |
| 201 | Tesfamariam et al. | Pre-ART nutritional status and its association with mortality in adult patients enrolled on ART at Fiche Hospital in North Shoa, Oromia region, Ethiopia: a retrospective cohort study. | 2016 | No data reported comparing CD4 vs. WHO stage |
| 202 | Verma et al. | Clinico-Social and Immunological Profile of Antiretroviral Naïve Children Living With HIV In Tertiary Care Hospital, Delhi | 2016 | No data reported comparing CD4 vs. WHO stage |
| 203 | Vinikoor et al. | Hepatitis B virus co-infection is associated with increased all-cause mortality among HIV-infected adults on tenofovir-disoproxil-fumarate containing antiretroviral therapy in Lusaka, Zambia | 2016 | It is a review, case-control study or conference abstract |
| 204 | Achappa et al. | Trends in CD4 count and who staging among newly diagnosed HIV patients attending an anti-retroviral therapy centre in tertiary care hospital | 2017 | It is a case-control study or conference abstract |
| 205 | Attinsounon et al. | Current Profile of New HIV Infections Among Adults in Northern Benin in 2016 | 2017 | No data reported comparing CD4 vs. WHO stage |
| 206 | Aung et al. | HIV care in Yangon, Myanmar; successes, challenges and implications for policy | 2017 | No data reported comparing CD4 vs. WHO stage |
| 207 | Bekalo et al. | Tuberculosis Incidence and Its Predictive Factors among Patients Receiving Antiretroviral Therapy in Dilla Hospital, Ethiopia | 2017 | No data reported comparing CD4 vs. WHO stage |
| 208 | Daranendran  et al. | Evaluation of Total Lymphocyte Count, Body Mass Index, Haemoglobin and Albumin levels as low cost surrogate markers for CD4 counts in assessing disease progression in HIV/AIDS-a Descriptive Study. | 2017 | No data reported comparing CD4 vs. WHO stage |
| 209 | Daye et al. | Facteurs associés à la dissociation immunovirologique chez les patients infectés par le VIH-1 sous traitement antirétroviral hautement actif au Centre de Traitement Ambulatoire (CTA) de Dakar | 2017 | No data reported comparing CD4 vs. WHO stage |
| 210 | Martin et al. | Late stage presentation of HIV-positive patients to antiretroviral outpatient clinic in Zambia | 2017 | No data reported comparing CD4 vs. WHO stage |
| 211 | Nampijja et al. | Dyslipidemia and its Correlates among HIV Infected Children on HAART Attending Mbarara Regional Referral Hospital | 2017 | No data reported comparing CD4 vs. WHO stage |
| 212 | Njom Nlend et al. | Predictors of mortality among HIV-infected children receiving highly active antiretroviral therapy. | 2017 | No data reported comparing CD4 vs. WHO stage |
| 213 | Ren et al. | Prognosis of HIV Patients Receiving Antiretroviral Therapy According to CD4 Counts: A Long-term Follow-up study in Yunnan, China | 2017 | No data reported comparing CD4 vs. WHO stage |
| 214 | Tadesse et al. | Cohort profile: improving treatment of HIV-infected Ethiopian children through better detection of treatment failure in southern Ethiopia. | 2017 | No data reported comparing CD4 vs. WHO stage |
| 215 | Tweya et al. | Characteristics and outcomes of older HIV-infected patients receiving antiretroviral therapy in Malawi: A retrospective observation cohort study | 2017 | No data reported comparing CD4 vs. WHO stage |
| 216 | Yassin et al. | Magnitude and predictors of antiretroviral treatment failure among HIV-infected children in Fiche and Kuyu hospitals, Oromia region, Ethiopia: a retrospective cohort study | 2017 | No data reported comparing CD4 vs. WHO stage |
| 217 | Yulian et al. | Effectiveness of one-stop service model of HIV testing and treatment in Zhongshan, Guangxi Zhuang Autonomous Region. | 2017 | No data reported comparing CD4 vs. WHO stage |
| 218 | Adal et al. | Associations of gender and serum total cholesterol with CD4+ T cell count and HIV RNA load in antiretroviral-naïve individuals in Addis Ababa | 2018 | No data reported comparing CD4 vs. WHO stage |
| 219 | Adal et al. | Malnutrition and lipid abnormalities in antiretroviral naive HIV-infected adults in Addis Ababa: A cross-sectional study. | 2018 | No data reported comparing CD4 vs. WHO stage |
| 220 | Bermudez-Aza et al. | Long-term clinical, immunological and virological outcomes of patients on antiretroviral therapy in southern Myanmar | 2018 | No data reported comparing CD4 vs. WHO stage |
| 221 | Butler et al. | Human immunodeficiency virus infection and older adults: A retrospective single-site cohort study from Johannesburg, South Africa | 2018 | No data reported comparing CD4 vs. WHO stage |
| 222 | Gesesew et al. | Late presentation for HIV care in Southwest Ethiopia in 2003–2015: prevalence, trend, outcomes and risk factors | 2018 | No data reported comparing CD4 vs. WHO stage |
| 223 | Gesesew et al. | Prevalence, trend, outcomes and risk factors for late presentation for HIV care in Ethiopia, 2003-2015 | 2018 | No data reported comparing CD4 vs. WHO stage |
| 224 | Gonzalez-Fernandez et al. | Advanced presentation among HIV/AIDS patients despite universal access to antiretroviral therapy in northern Mexico | 2018 | It is a case-control study or conference abstract |
| 225 | Gunda et al. | Prevalence and Risk Factors of Active TB among Adult HIV Patients Receiving ART in Northwestern Tanzania: A Retrospective Cohort Study. | 2018 | No data reported comparing CD4 vs. WHO stage |
| 226 | Inamdar et al. | Age and opportunistic infections: prevalence and predictors among older people living with HIV. | 2018 | No data reported comparing CD4 vs. WHO stage |
| 227 | Khamis et al. | Epidemiological and Clinical Characteristics of HIV Infected Patients at a Tertiary Care Hospital in Oman | 2018 | No data reported comparing CD4 vs. WHO stage |
| 228 | Liu et al. | Effect of baseline CD/T cell count on drop-out of antiretroviral therapy in HIV infected persons in Guangxi Zhuang Autonomous Region, 2008-2015 | 2018 | It is a review, case-control study or conference abstract |
| 229 | Luma et al. | Late presentation to HIV/AIDS care at the Douala general hospital, Cameroon: its associated factors, and consequences | 2018 | No data reported comparing CD4 vs. WHO stage |
| 230 | Mardarescu et al. | Romania in HIV/AIDS numbers 1985 to 2017: Cascade of care in HIV/AIDS infection | 2018 | It is a case-control study or conference abstract |
| 231 | Mwihaki et al. | Correlates of Retention in Care Among HIV Infected Adolescents and Young Adults Enrolled in an HIV Program in Kenya | 2018 | It is a case-control study or conference abstract |
| 232 | Ngom et al. | ART initiation in an outpatient treatment center in Dakar, Senegal: A retrospective cohort analysis (1998-2015) | 2018 | No data reported comparing CD4 vs. WHO stage |
| 233 | Sajmi Shaji et al. | Study of Functioning of Thyroid in HIV Infected Patients | 2018 | No data reported comparing CD4 vs. WHO stage |
| 234 | Shaji et al. | Study of Functioning of Thyroid in HIV Infected Patients. | 2018 | No data reported comparing CD4 vs. WHO stage |
| 235 | Siika et al. | Late Presentation with HIV in Africa: Phenotypes, Risk, and Risk Stratification in the REALITY Trial | 2018 | The paper does not use a CD4 threshold equal to or less than 200 |
| 236 | Ssebutinde  et al. | Effect of age at initiation of antiretroviral therapy on treatment outcomes; A retrospective cohort study at a large HIV clinic in southwestern Uganda | 2018 | No data reported comparing CD4 vs. WHO stage |
| 237 | Tang et al. | Late for testing, early for antiretroviral therapy, less likely to die": Results from a large HIV cohort study in China, 2006-2014 | 2018 | No data reported comparing CD4 vs. WHO stage |
| 238 | Yendewa et al. | High Prevalence of Late-Stage Disease in Newly Diagnosed Human Immunodeficiency Virus Patients in Sierra Leone | 2018 | No data reported comparing CD4 vs. WHO stage |
| 239 | Aksak-Wazs et al. | Factors influencing late presentation into care of HIV disease | 2019 | It is a case-control study or conference abstract |
| 240 | Angdembe et al. | Predictors of mortality in adult people living with HIV on antiretroviral therapy in Nepal: A retrospective cohort study, 2004-2013 | 2019 | No data reported comparing CD4 vs. WHO stage |
| 241 | Bhatta et al. | Life expectancy and disparities in survival among HIV-infected people receiving antiretroviral therapy: an observational cohort study in Kathmandu, Nepal. | 2019 | No data reported comparing CD4 vs. WHO stage |
| 242 | Bilcha  et al. | Predictors of Time to First Line Antiretroviral Treatment Failure among Adult Patients Living with HIV in Public Health Facilities of Arba Minch Town, Southern Ethiopia. | 2019 | No data reported comparing CD4 vs. WHO stage |
| 243 | Hidalgo et al. | A comparison of baseline conditions and time to initiation of ART between 2005, 2011 and 2016 at an HIV treatment centre in Lima, Peru | 2019 | No data reported comparing CD4 vs. WHO stage |
| 244 | Kim et al. | Late diagnosis and delayed presentation in Korean HIV/AIDS cohort | 2019 | It is a case-control study or conference abstract |
| 245 | Kiragga et al. | A decade of antiretroviral therapy in Uganda: what are the emerging causes of death? | 2019 | No data reported comparing CD4 vs. WHO stage |
| 246 | Larsen et al. | Timing and Predictors of Initiation on Antiretroviral Therapy Among Newly Diagnosed HIV Infected Persons in South Africa | 2019 | No data reported comparing CD4 vs. WHO stage |
| 247 | Lifson et al. | Advanced HIV Disease among Males and Females Initiating HIV Care in Rural Ethiopia. | 2019 | No data reported comparing CD4 vs. WHO stage |
| 248 | Mbalabu et al. | Epidemiological and clinical profile of HIV-infected patients at initiation of haart in Lubumbashi settings, the Democratic Republic of the Congo | 2019 | It is a case-control study or conference abstract |
| 249 | McKenzie et al. | Poor clinical outcomes in HIV-infected children who start antiretroviral therapy at an older age | 2019 | It is a case-control study or conference abstract |
| 250 | Nkhoma et al. | Is symptom prevalence and burden associated with HIV treatment status and disease stage among adult HIV outpatients in Kenya? A cross-sectional self-report study. | 2019 | No data reported comparing CD4 vs. WHO stage |
| 251 | Nsanzimana et al. | Retention in care and virological failure among adult HIV+ patients on second-line ART in Rwanda: a national representative study. | 2019 | No data reported comparing CD4 vs. WHO stage |
| 252 | Adeola et al. | Effect of Baseline Symptom Manifestations on Retention in Care and Treatment among HIV-Infected Patients in Nigeria | 2020 | No data reported comparing CD4 vs. WHO stage |
| 253 | Alemu et al. | Incidence and predictors of extrapulmonary tuberculosis among people living with Human Immunodeficiency Virus in Addis Ababa, Ethiopia: A retrospective cohort study | 2020 | No data reported comparing CD4 vs. WHO stage |
| 254 | Andualem et al. | Progression of HIV Disease Among Patients on ART in Ethiopia: Application of Longitudinal Count Models. | 2020 | No data reported comparing CD4 vs. WHO stage |
| 255 | Arinze et al. | Immunodeficiency at Antiretroviral Therapy Start: Five-Year Adult Data (2012-2017) Based on Evolving National Policies in Rural Mozambique. | 2020 | No data reported comparing CD4 vs. WHO stage |
| 256 | Birhan et al. | Predictors of CD4 count changes over time among children who initiated highly active antiretroviral therapy in Ethiopia | 2020 | No data reported comparing CD4 vs. WHO stage |
| 257 | Chanto et al. | Causes of Hospitalization and Death among Newly Diagnosed HIV-Infected Adults in Thailand | 2020 | No data reported comparing CD4 vs. WHO stage |
| 258 | Desmonde et al. | Time-varying age- and CD4-stratified rates of mortality and WHO stage 3 and stage 4 events in children, adolescents and youth 0 to 24 years living with perinatally acquired HIV, before and after antiretroviral therapy initiation in the paediatric IeDEA GlobalCohort Consortium | 2020 | No data reported comparing CD4 vs. WHO stage |
| 259 | Haas et al. | Excess mortality associated with mental illness in people living with HIV in Cape Town, South Africa: a cohort study using linked electronic health records. | 2020 | No data reported comparing CD4 vs. WHO stage |
| 260 | Kalinjuma et al. | Prospective assessment of loss to follow-up: incidence and associated factors in a cohort of HIV-positive adults in rural Tanzania. | 2020 | No data reported comparing CD4 vs. WHO stage |
| 261 | Kiwanuka et al. | Determinants of loss to follow-up among HIV positive patients receiving antiretroviral therapy in a test and treat setting: A retrospective cohort study in Masaka, Uganda. | 2020 | No data reported comparing CD4 vs. WHO stage |
| 262 | Michealis et al. | Late diagnosis of human immunodeficiency virus infection is linked to higher rates of epilepsy in children in the Eastern Cape of South Africa. | 2020 | No data reported comparing CD4 vs. WHO stage |
| 263 | Mugusi et al. | Impact of Population and Pharmacogenetics Variations on Efavirenz Pharmacokinetics and Immunologic Outcomes During Anti-Tuberculosis Co-Therapy: A Parallel Prospective Cohort Study in Two Sub-Sahara African Populations | 2020 | No data reported comparing CD4 vs. WHO stage |
| 264 | Nasuuna et al. | Reduction in Baseline CD4 Count Testing Following Human Immunodeficiency Virus "Treat All" Adoption in Uganda | 2020 | No data reported comparing CD4 vs. WHO stage |
| 265 | Patsis et al. | Lower rates of ART initiation and decreased retention among ART-naive patients who consume alcohol enrolling in HIV care and treatment programs in Kenya and Uganda. | 2020 | No data reported comparing CD4 vs. WHO stage |
| 266 | Suryana et al. | The association between WHO clinical stage and other risk factors with pulmonary tuberculosis among people living with HIV/AIDS: A cross-sectional study | 2020 | No data reported comparing CD4 vs. WHO stage |
| 267 | Widiyanti et al. | Body mass index increases CD4+ count in HIV/AIDS patients on first-line therapy. | 2020 | No data reported comparing CD4 vs. WHO stage |
| 268 | Zheng Wu et al. | Survival time and influence factors of HAART in Hubei, 2008-2018. | 2020 | No data reported comparing CD4 vs. WHO stage |
| 269 | Adedimeji et al. | Trends in demographic and clinical characteristics and initiation of antiretroviral therapy among adult patients enrolling in HIV care in the Central Africa International epidemiology Database to Evaluate AIDS (CA-IeDEA) 2004 to 2018 | 2021 | No data reported comparing CD4 vs. WHO stage |
| 270 | Bayarsaikhan et al. | Survival, CD4 T lymphocyte count recovery and immune reconstitution pattern during the first-line combination antiretroviral therapy in patients with HIV-1 infection in Mongolia. | 2021 | No data reported comparing CD4 vs. WHO stage |
| 271 | Belay et al. | Predictors of current cd4+ t-cell count among women of reproductive age on antiretroviral therapy in public hospitals, southwest ethiopia | 2021 | No data reported comparing CD4 vs. WHO stage |
| 272 | Benzekri et al. | Impact of Traditional Healers on the HIV Care Cascade in Senegal, West Africa: A Longitudinal Study | 2021 | No data reported comparing CD4 vs. WHO stage |
| 273 | Dakum et al. | Prevalence and risk factors for obesity among elderly patients living with HIV/AIDS in a low-resource setting | 2021 | No data reported comparing CD4 vs. WHO stage |
| 274 | Dobe et al. | Earlier antiretroviral initiation is independently associated with better arterial stiffness in children living with perinatally acquired HIV with sustained viral suppression in Mozambique | 2021 | No data reported comparing CD4 vs. WHO stage |
| 275 | LiuHong et al. | Death and attrition among HIV-infected patients receiving initial antiretroviral therapy in Qinzhou, Guangxi, 2008-2018 | 2021 | No data reported comparing CD4 vs. WHO stage |
| 276 | Ngongo et al. | Longitudinal analysis of sociodemographic, clinical and therapeutic factors of HIV-infected individuals in Kinshasa at antiretroviral therapy initiation during 2006-2017. | 2021 | No data reported comparing CD4 vs. WHO stage |
| 277 | Onoya et al. | Incidence and predictors of sexually transmitted infections among adult HIV-positive patients receiving antiretroviral therapy at Themba Lethu HIV clinic in Johannesburg, South Africa | 2021 | No data reported comparing CD4 vs. WHO stage |
| 278 | Rukundo et al. | Effect of suicidality on clinical and behavioural outcomes in HIV positive adults in Uganda | 2021 | No data reported comparing CD4 vs. WHO stage |
| 279 | Shamu et al. | Treatment outcomes in HIV infected patients older than 50 years attending an HIV clinic in Harare, Zimbabwe: A cohort study. | 2021 | No data reported comparing CD4 vs. WHO stage |
| 280 | Traoré et al. | Socio-demographic, clinical, and therapeutic aspects of adolescents treated and followed for HIV infection at the Hospital Gabriel Toure paediatric center | 2021 | No data reported comparing CD4 vs. WHO stage |
| 281 | Vanobberghen et al. | Mortality Rate in a Cohort of People Living With HIV in Rural Tanzania After Accounting for Unseen Deaths Among Those Lost to Follow-up | 2021 | No data reported comparing CD4 vs. WHO stage |
| 282 | Walubo et al. | Clinical Characteristics of Children with HIV Initiated on Antiretroviral Treatment at HIV Clinics in Bloemfontein, South Africa. | 2021 | No data reported comparing CD4 vs. WHO stage |
| 283 | Zeru et al. | Prevalence and associated factors of HIV-TB co-infection among HIV patients: a retrospective Study. | 2021 | No data reported comparing CD4 vs. WHO stage |
| 284 | Afrashteh et al. | Factors Associated with Baseline CD4 Cell Counts and Advanced HIV Disease among Male and Female HIV-Positive Patients in Iran: A Retrospective Cohort Study | 2022 | No data reported comparing CD4 vs. WHO stage |
| 285 | Agarwal et al. | A cross-sectional study of demographic and clinical profile of HIV patients at ART center of tertiary care hospital | 2022 | No data reported comparing CD4 vs. WHO stage |
| 286 | Amour et al. | Adherence to Antiretroviral Therapy by Medication Possession Ratio and Virological Suppression among Adolescents and Young Adults Living with HIV in Dar es Salaam, Tanzania | 2022 | No data reported comparing CD4 vs. WHO stage |
| 287 | Byers et al. | Comparison of predictors for early and late mortality in adults commencing HIV antiretroviral therapy in Zimbabwe: a retrospective cohort study. | 2022 | No data reported comparing CD4 vs. WHO stage |
| 288 | Fokam et al. | Archiving of mutations in HIV-1 cellular reservoirs among vertically infected adolescents is contingent with clinical stages and plasma viral load: Evidence from the EDCTP-READY study | 2022 | No data reported comparing CD4 vs. WHO stage |
| 289 | Hong et al. | Predictors of loss to follow-up from HIV antiretroviral therapy in Namibia | 2022 | No data reported comparing CD4 vs. WHO stage |
| 290 | Kabarambi et al. | Determinants and reasons for switching anti-retroviral regimen among HIV-infected youth in a large township of South Africa (2002-2019). | 2022 | No data reported comparing CD4 vs. WHO stage |
| 291 | Katende et al. | Antiretroviral therapy initiation and outcomes of hospitalized HIV-infected patients in Uganda-An evaluation of the HIV test and treat strategy. | 2022 | No data reported comparing CD4 vs. WHO stage |
| 292 | Kotey et al. | Chronic comorbidities in persons living with HIV within three years of exposure to antiretroviral therapy at Pantang Antiretroviral Center in Ghana: a retrospective study | 2022 | No data reported comparing CD4 vs. WHO stage |
| 293 | Mollel et al. | Causes of death and associated factors over a decade of follow-up in a cohort of people living with HIV in rural Tanzania. | 2022 | No data reported comparing CD4 vs. WHO stage |
| 294 | Shiferaw et al. | Incidence and predictors of loss to follow-up among adult HIV-infected patients taking antiretroviral therapy at North Shewa zone public Hospitals, Northeast Ethiopia: A retrospective follow-up study. | 2022 | No data reported comparing CD4 vs. WHO stage |
| 295 | GebreEyesus et al. | Sleep quality and associated factors among adult people living with HIV on follow-up at Dessie Town Governmental Health Facilities Antiretroviral Therapy Clinics, Northeast, Ethiopia, 2020, a multicenter cross-sectional study. | 2023 | No data reported comparing CD4 vs. WHO stage |
| 296 | Gebrerufael et al. | Predictors associated with CD4 cell count changes over time among HIV-infected children on anti-retroviral therapy follow-up in Mekelle General Hospital, Northern Ethiopia, 2019: a retrospective longitudinal study | 2023 | No data reported comparing CD4 vs. WHO stage |
| 297 | GuoXian et al. | Correlation analysis of baseline indicators of HIV infected/aids patients and immune function reconstruction after antiretroviral therapy. | 2023 | No data reported comparing CD4 vs. WHO stage |
| 298 | Hong et al. | Immunological CD4+ T-cell recovery and prognostic influencing factors in elderly HIV/AIDS patients of different genders after antiviral therapy. | 2023 | No data reported comparing CD4 vs. WHO stage |
| 299 | Hoyos-Pulgarín et al. | Clinical and demographic characteristics, and problems related to medication in patients with 50 or more years with recent diagnosis of HIV in Medellin, Colombia | 2023 | No data reported comparing CD4 vs. WHO stage |
| 300 | Mbatia et al. | Interruptions in treatment among adults on anti-retroviral therapy before and after test-and-treat policy in Tanzania | 2023 | The paper does not use a CD4 threshold equal to or less than 200 |
| 301 | Mengistu et al. | Antiretroviral therapy regimen modification rates and associated factors in a cohort of HIV/AIDS patients in Asmara, Eritrea: a 16-year retrospective analysis. | 2023 | No data reported comparing CD4 vs. WHO stage |
| 302 | Mugenyi et al. | Effect of the "universal test and treat" policy on the characteristics of persons registering for HIV care and initiating antiretroviral therapy in Uganda. | 2023 | No data reported comparing CD4 vs. WHO stage |
| 303 | Muhie et al. | Predictors for CD4 cell count and hemoglobin level with survival time to default for HIV positive adults under ART treatment at University of Gondar Comprehensive and Specialized Hospital, Ethiopia. | 2023 | No data reported comparing CD4 vs. WHO stage |
| 304 | Ojiambo et al. | Socio-demographic and clinical characteristics associated with retention in care among adults living with HIV and severe mental illness and reasons for loss to follow-up in Uganda: a mixed-methods study | 2023 | No data reported comparing CD4 vs. WHO stage |
| 305 | Ouedraogo et al. | Transition of adolescents living with HIV from pediatric to adult care, a retrospective 12-year Single Center Study from the Sahel Region in West-Africa | 2023 | No data reported comparing CD4 vs. WHO stage |
| 306 | Dagnaw et al. | Time to develop adverse drug reactions and associated factors among children HIV positive patients on antiretroviral treatment in Northwest Amhara Specialized Hospitals: Retrospective cohort study, 2022 | 2024 | No data reported comparing CD4 vs. WHO stage |
| 307 | Muthuka et al. | Paradoxical and Unmasking HIV Immune Reconstitution Inflammation Syndrome in Antiretroviral-Naive Pregnant Women: A Prospective Cohort Study. | 2024 | No data reported comparing CD4 vs. WHO stage |
| 308 | Oboho et al. | Advanced HIV disease in East Africa and Nigeria, in The African Cohort Study. | 2024 | No data reported comparing CD4 vs. WHO stage |
| 309 | Pu et al. | Survival analysis of PLWHA undergoing combined antiretroviral therapy: exploring long-term prognosis and influencing factors | 2024 | No data reported comparing CD4 vs. WHO stage |
| 310 | Secor et al. | Does HIV index testing bring patients into treatment at earlier stages of HIV disease? Results from a retrospective study in Ukraine. | 2024 | No data reported comparing CD4 vs. WHO stage |

# Supplementary Figure 1. Summary receiver operator characteristic curve for WHO clinical stage 3 or 4 classification to detect advanced HIV disease


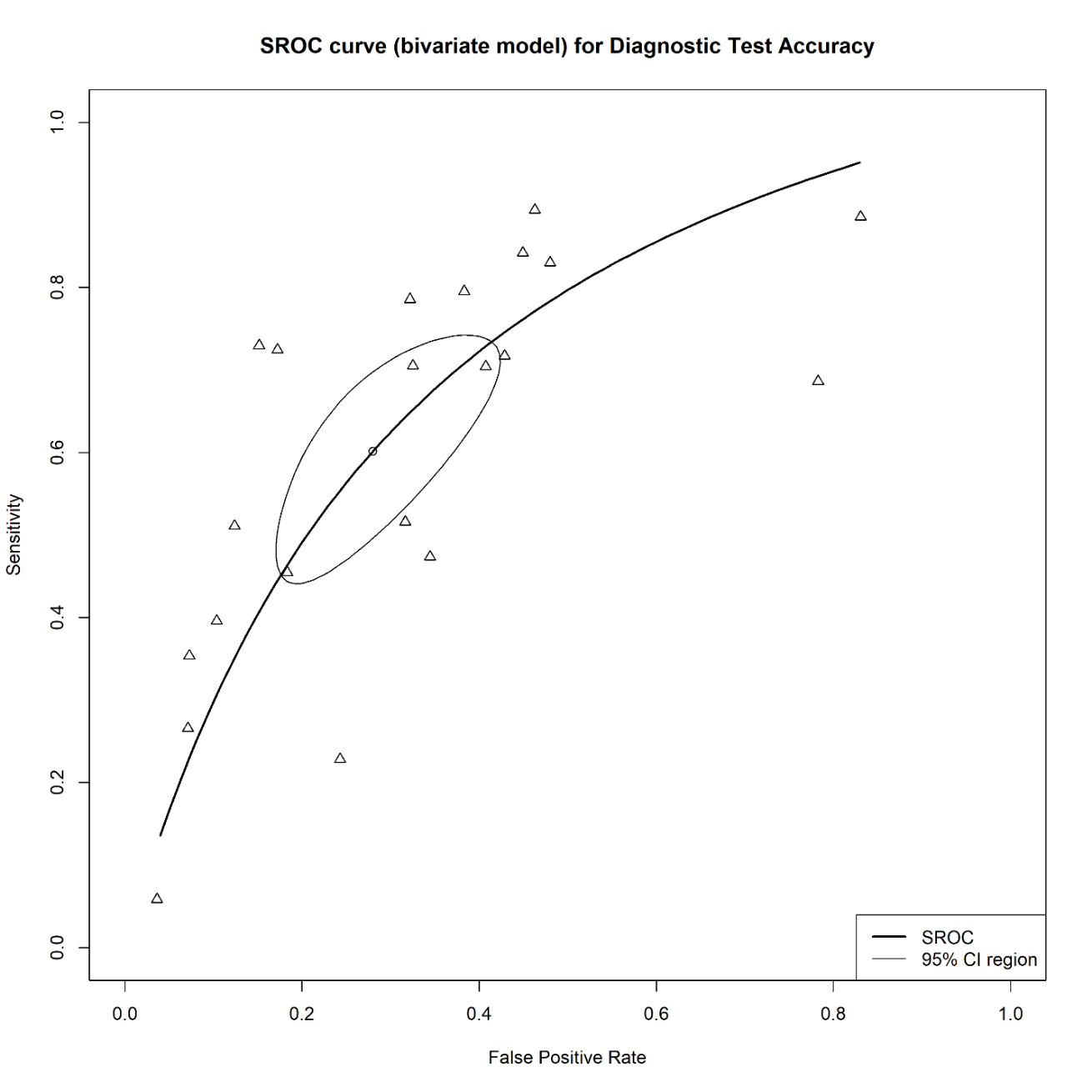


SROC = summary receiver operator characteristic, CI = confidence interval

# Supplementary Figure 2. Risk of bias assessment

| **Study** | Was a consecutive or random sample of patients enrolled? | Was a case-control design avoided? | Did the study avoid inappropriate exclusions? | Could the selection of patients have introduced bias? | Is there concern that the included patients do not match the review question? | Were the index test (WHO staging) results interpreted without knowledge of the result of the reference standard (CD4 count)? | If a threshold was used, was it pre-specified? | Could the conduct or interpretation of the index test (WHO staging) have introduced bias? | Is there concern that the index test (WHO staging), its conduct, or interpretation differ from the review question? | Is the reference standard (CD4 count) likely to correctly classify the target condition (Advanced HIV disease)? | Were the reference standard (CD4 count) results interpreted without knowledge of the results of the index test (WHO staging)? | Could the reference standard (CD4 count), its conduct, or its interpretation have introduced bias? | Is there concern that the target condition (stage 3 or 4 HIV disease) as defined by the reference standard (CD4 count) does not match the review question? | Was there an appropriate interval between index test (WHO staging) and reference standard (CD4 count)? | Did all patients receive a reference standard (CD4 count)? | Did patients receive the same reference standard (CD4 count)? | Were all patients included in the analysis? | Could the patient flow have introduced bias? | Overall judgment of risk of bias |
| --- | --- | --- | --- | --- | --- | --- | --- | --- | --- | --- | --- | --- | --- | --- | --- | --- | --- | --- | --- |
| **Aregay ^1^** | **Unclear** | **Yes** | **Yes** | **Low** | **Low** | **Unclear** | **Yes** | **Low** | **Low** | **Yes** | **Unclear** | **Low** | **Low** | **Unclear** | **Yes** | **Yes** | **Yes** | **Low** | **Low** |
| **Balde ^2^** | **Yes** | **Yes** | **Yes** | **Low** | **Low** | **Unclear** | **Yes** | **Low** | **Low** | **Yes** | **Unclear** | **Unclear** | **Low** | **Unclear** | **Yes** | **Yes** | **Yes** | **Low** | **Low** |
| **Boniphace ^3^** | **Yes** | **Yes** | **Yes** | **Low** | **Low** | **Unclear** | **Yes** | **Unclear** | **Low** | **Yes** | **Unclear** | **Low** | **Low** | **Unclear** | **Yes** | **Yes** | **No** | **Unclear** | **Moderate** |
| **Carter ^4^** | **Unclear** | **Yes** | **Yes** | **Low** | **Unclear** | **Unclear** | **Yes** | **Low** | **Low** | **Yes** | **Unclear** | **Unclear** | **Low** | **Yes** | **No** | **Yes** | **No** | **High** | **High** |
| **Ebonyi ^5^** | **Yes** | **Yes** | **Yes** | **Low** | **Low** | **Unclear** | **Yes** | **Low** | **Low** | **Yes** | **Unclear** | **Low** | **Low** | **Yes** | **Yes** | **Yes** | **Yes** | **Low** | **Low** |
| **Edathodu ^6^** | **Yes** | **Yes** | **No** | **High** | **Low** | **Yes** | **No** | **Low** | **Low** | **Yes** | **Yes** | **Low** | **Low** | **No** | **Yes** | **Yes** | **Yes** | **Low** | **High** |
| **French ^7^** | **Yes** | **Yes** | **Yes** | **Low** | **Low** | **Yes** | **Yes** | **High** | **High** | **Yes** | **Yes** | **Low** | **Low** | **Unclear** | **Yes** | **Yes** | **Yes** | **Low** | **Moderate** |
| **Gautam ^8^** | **Unclear** | **Yes** | **Unclear** | **Unclear** | **Low** | **Unclear** | **Yes** | **Unclear** | **Low** | **Yes** | **Unclear** | **Unclear** | **Low** | **Unclear** | **Yes** | **Yes** | **Yes** | **Low** | **Moderate** |
| **Haider ^9^** | **Yes** | **Yes** | **Yes** | **Low** | **Low** | **Unclear** | **No** | **Unclear** | **Unclear** | **Yes** | **Unclear** | **Unclear** | **Low** | **Unclear** | **Yes** | **Yes** | **Yes** | **Low** | **Moderate** |
| **Ilovi ^10^** | **Unclear** | **Yes** | **No** | **High** | **Low** | **Yes** | **Yes** | **High** | **Low** | **Yes** | **Unclear** | **Low** | **Low** | **No** | **Yes** | **Yes** | **Yes** | **Low** | **High** |
| **Ingole ^11^** | **Unclear** | **Yes** | **Yes** | **Unclear** | **Unclear** | **Unclear** | **Yes** | **Low** | **Low** | **Yes** | **Unclear** | **Low** | **Low** | **Unclear** | **Yes** | **Yes** | **Yes** | **Low** | **Moderate** |
| **Jaffar ^12^** | **Yes** | **Yes** | **Yes** | **Low** | **Low** | **Yes** | **Yes** | **Unclear** | **Low** | **Yes** | **Unclear** | **Low** | **Low** | **Unclear** | **Yes** | **Yes** | **Yes** | **Unclear** | **Moderate** |
| **Kagaayi ^13^** | **Unclear** | **Yes** | **Unclear** | **Unclear** | **Low** | **Unclear** | **Yes** | **Low** | **Low** | **Yes** | **Yes** | **Low** | **Low** | **Unclear** | **Yes** | **Yes** | **Yes** | **Low** | **Moderate** |
| **Kassa ^14^** | **No** | **Yes** | **Unclear** | **High** | **High** | **Unclear** | **No** | **Low** | **Low** | **Yes** | **Unclear** | **Unclear** | **Low** | **Unclear** | **Yes** | **Yes** | **No** | **High** | **High** |
| **Klotz ^15^** | **Yes** | **Yes** | **Yes** | **Low** | **Low** | **Unclear** | **Unclear** | **Unclear** | **Unclear** | **Yes** | **Unclear** | **Low** | **Low** | **Unclear** | **No** | **Yes** | **Unclear** | **Unclear** | **Moderate** |
| **Lebelonyane ^16^** | **Yes** | **Yes** | **Yes** | **Low** | **Low** | **Unclear** | **Yes** | **Low** | **Low** | **Yes** | **Unclear** | **Unclear** | **Low** | **Unclear** | **Yes** | **Yes** | **Yes** | **Low** | **Moderate** |
| **Morpeth ^17^** | **Yes** | **Yes** | **Unclear** | **Unclear** | **Low** | **Unclear** | **Yes** | **Unclear** | **Unclear** | **Yes** | **Yes** | **Low** | **Low** | **Unclear** | **Unclear** | **Yes** | **Yes** | **Unclear** | **Moderate** |
| **Nyagaka ^18^** | **Unclear** | **Yes** | **Unclear** | **Unclear** | **Low** | **Unclear** | **Unclear** | **Unclear** | **Unclear** | **Yes** | **Unclear** | **Unclear** | **Low** | **Unclear** | **Yes** | **Unclear** | **Yes** | **Unclear** | **Moderate** |
| **Oudenhoven ^19^** | **Yes** | **Yes** | **Yes** | **Low** | **Low** | **Unclear** | **Yes** | **Unclear** | **Low** | **Yes** | **Unclear** | **Low** | **Low** | **Unclear** | **Unclear** | **Yes** | **No** | **Unclear** | **Moderate** |
| **Reda ^20^** | **Yes** | **Yes** | **Yes** | **Low** | **Low** | **Unclear** | **No** | **Unclear** | **Unclear** | **Yes** | **Unclear** | **Unclear** | **Low** | **Unclear** | **No** | **Yes** | **No** | **Low** | **High** |
| **Sempa ^21^** | **Yes** | **Yes** | **Unclear** | **Unclear** | **Low** | **Unclear** | **Yes** | **Unclear** | **Low** | **Yes** | **Unclear** | **Unclear** | **Low** | **Unclear** | **Yes** | **Yes** | **Yes** | **Unclear** | **Moderate** |
| **Tassie ^22^** | **Yes** | **Yes** | **Unclear** | **Unclear** | **Low** | **Unclear** | **Yes** | **Unclear** | **Low** | **Yes** | **Unclear** | **Unclear** | **Low** | **Unclear** | **Yes** | **Yes** | **Yes** | **Unclear** | **Moderate** |
| **Torpey ^23^** | **Yes** | **Yes** | **Yes** | **Low** | **Low** | **Unclear** | **Yes** | **Unclear** | **Low** | **Yes** | **Unclear** | **Unclear** | **Low** | **Unclear** | **Yes** | **Yes** | **No** | **Low** | **Moderate** |
| **Visser ^24^** | **Yes** | **Yes** | **Yes** | **Unclear** | **Low** | **Unclear** | **Yes** | **Unclear** | **Low** | **Yes** | **Unclear** | **Low** | **Low** | **Unclear** | **Unclear** | **Yes** | **No** | **High** | **High** |
| **McGrath ^25^** | **Yes** | **Yes** | **Yes** | **Low** | **Low** | **Unclear** | **No** | **Unclear** | **Low** | **Yes** | **Unclear** | **Unclear** | **Low** | **Unclear** | **No** | **Yes** | **No** | **High** | **High** |

# Supplementary Figure 3. Funnel plots for sensitivity (A) and specificity (B)


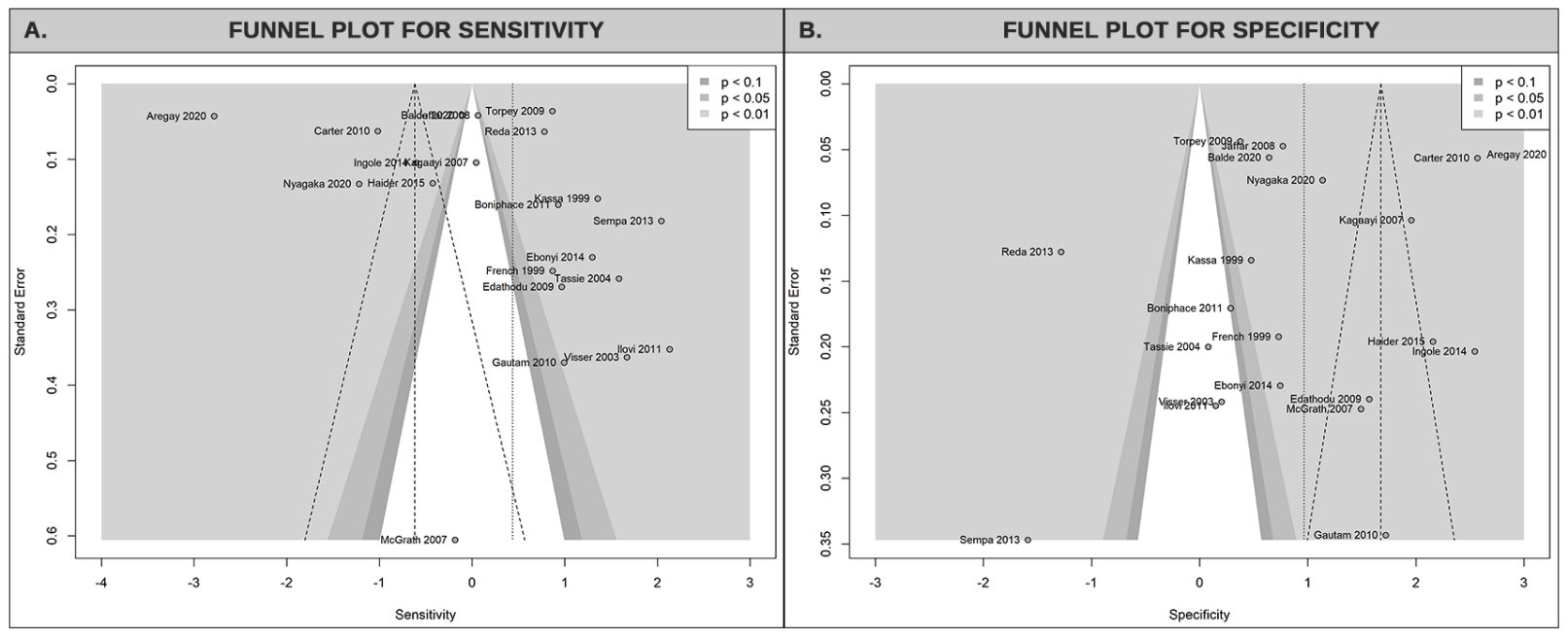


# Supplementary figure 4. Forest plots for sensitivity and specificity of WHO clinical stage 3 or 4 classification for detection of advanced HIV disease stratified by period

**
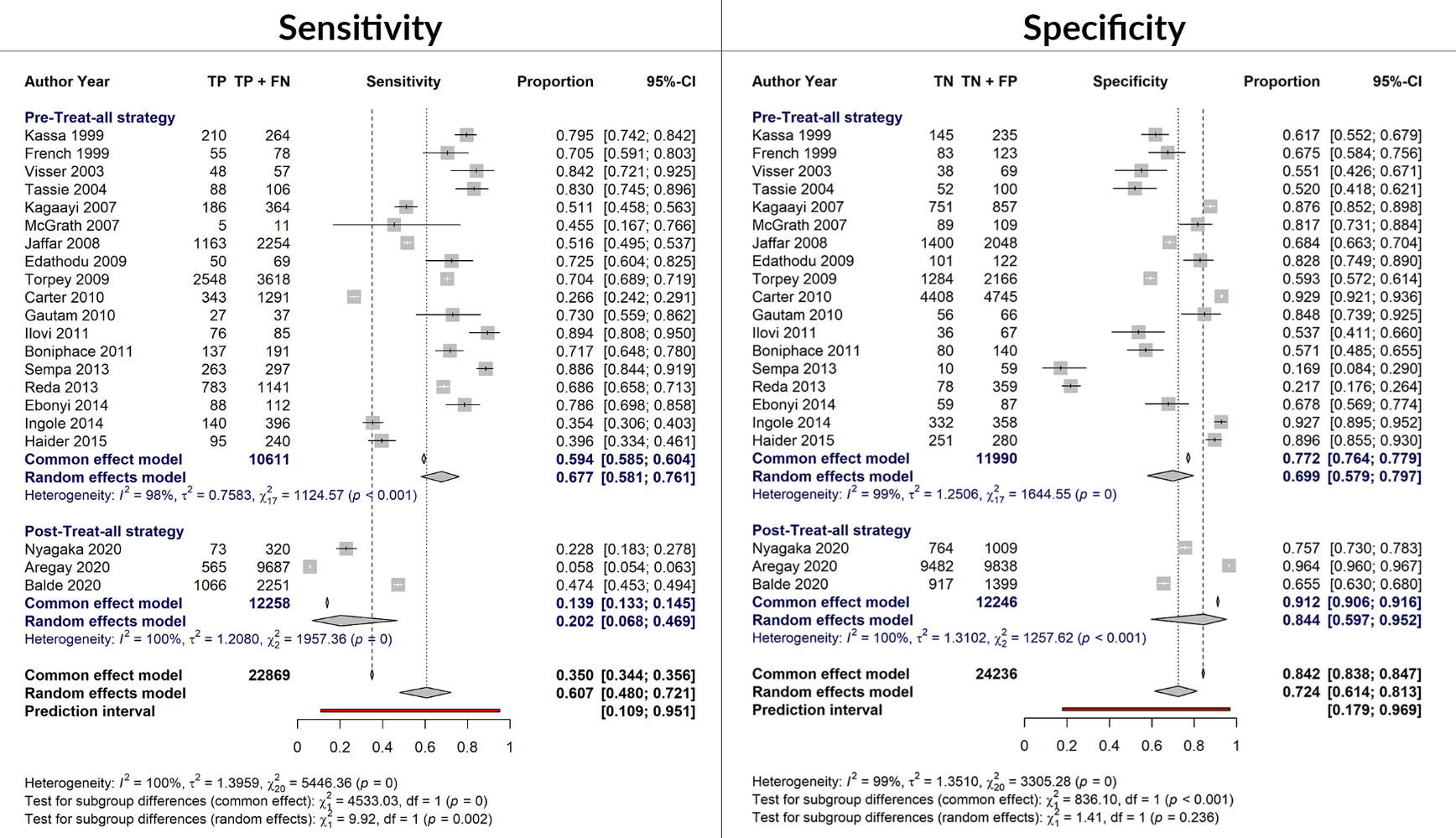
**

TP = true positive; FN = false negative; TN = true negative; FP = false positive

# Supplementary figure 5. Forest plots for sensitivity and specificity of WHO clinical stage 3 or 4 classification for detection of advanced HIV disease stratified by period


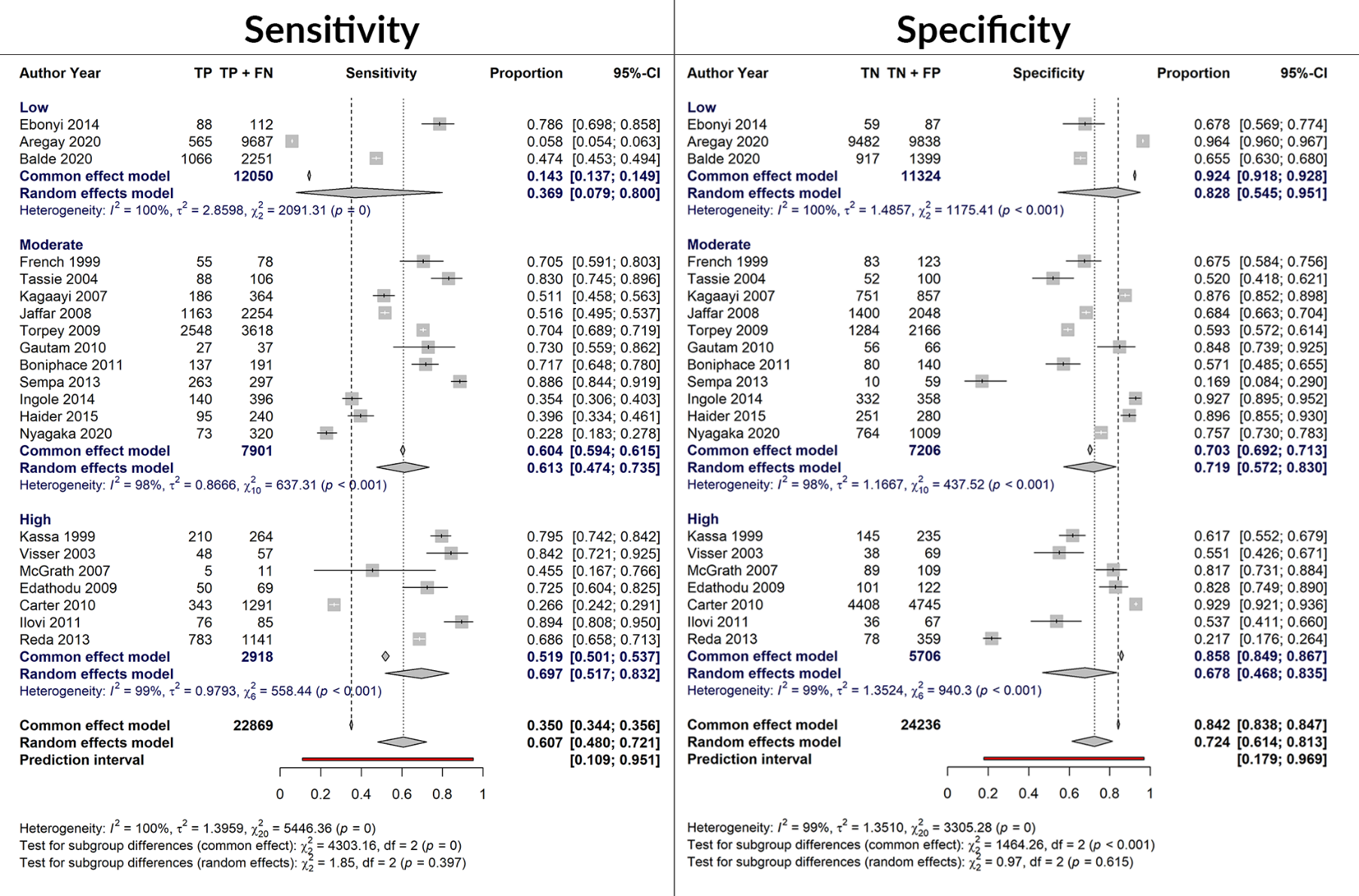


TP = true positive; FN = false negative; TN = true negative; FP = false positive

# References

1. Aregay AD, Kidane KM, Aregay AB, Fenta KA, Woldegebriel AG, Godefay H, et al. Prediction of CD4 T-Lymphocyte Count Using WHO Clinical Staging among ART-Naïve HIV-Infected Adolescents and Adults in Northern Ethiopia: A Retrospective Study. AIDS Res Treat. 2020;2020:2163486.

2. Baldé A, Lièvre L, Maiga AI, Diallo F, Maiga IA, Costagliola D, et al. Re-engagement in care of people living with HIV lost to follow-up after initiation of antiretroviral therapy in Mali: Who returns to care? PloS One. 2020;15(9):e0238687.

3. Boniphace I, Omari M, Susan Fred R, Ferdinand M, Marcel T. HIV/AIDS Clinical Manifestations and their Implication for Patient Clinical Staging in Resource Limited Settings in Tanzania. Open AIDS J. 2011;5:9–16.

4. Carter RJ, Dugan K, El-Sadr WM, Myer L, Otieno J, Pungpapong N, et al. CD4+ cell count testing more effective than HIV disease clinical staging in identifying pregnant and postpartum women eligible for antiretroviral therapy in resource-limited settings. J Acquir Immune Defic Syndr 1999. 2010 Nov;55(3):404–10.

5. Ebonyi AO, Agbaji OO, Anejo-Okopi JA, Oguche S, Agaba PA, Sagay SA, et al. Factors Associated with a Low CD4 Count among HIV-1 Infected Patients at Enrolment into HAART in Jos, Nigeria. J Adv Med Med Res [Internet]. 2014 Feb 18 [cited 2024 Dec 12];2536–45. Available from: https://journaljammr.com/index.php/JAMMR/article/view/1694

6. Edathodu J, Ali B, Alrajhi AA. CD4 validation for the World Health Organization classification and clinical staging of HIV/AIDS in a developing country. Int J Infect Dis IJID Off Publ Int Soc Infect Dis. 2009 Mar;13(2):243–6.

7. French N, Mujugira A, Nakiyingi J, Mulder D, Janoff EN, Gilks CF. Immunologic and clinical stages in HIV-1-infected Ugandan adults are comparable and provide no evidence of rapid progression but poor survival with advanced disease. J Acquir Immune Defic Syndr 1999. 1999 Dec 15;22(5):509–16.

8. Gautam H, Saini S, Bhalla P, Singh T. Use of total lymphocyte count to predict absolute CD4 count in HIV-seropositive cases. J Int Assoc Physicians AIDS Care Chic Ill 2002. 2010;9(5):292–5.

9. Haider S, Kumar V, Singh SB, Sunderam S. A Study on Socio-demographic Profile and CD4 Count of HIV Infected Patients Attending ART Centre RIMS, Ranchi. Healthline [Internet]. 2015 [cited 2024 Dec 12];6(1):12–7. Available from: https://journals.indexcopernicus.com/publication/792101/Haider-Shamim-A-Study-on

10. Ilovi CS, Lule GN, Obel AO, Irimu HM. Correlation of WHO clinical staging with CD4 counts in adult HIV/AIDS patients at Kenyatta National Hospital, Nairobi. East Afr Med J. 2011 Feb;88(2):65–70.

11. Ingole N, Nataraj G, Mehta P, Paranjpe S, Sarkate P. CD4 counts in laboratory monitoring of HIV disease--experience from western India. J Int Assoc Provid AIDS Care. 2014;13(4):324–7.

12. Jaffar S, Birungi J, Grosskurth H, Amuron B, Namara G, Nabiryo C, et al. Use of WHO clinical stage for assessing patient eligibility to antiretroviral therapy in a routine health service setting in Jinja, Uganda. AIDS Res Ther. 2008 Feb 28;5:4.

13. Kagaayi J, Makumbi F, Nakigozi G, Wawer MJ, Gray RH, Serwadda D, et al. WHO HIV clinical staging or CD4 cell counts for antiretroviral therapy eligibility assessment? An evaluation in rural Rakai district, Uganda. AIDS Lond Engl. 2007 May 31;21(9):1208–10.

14. Kassa E, Rinke de Wit TF, Hailu E, Girma M, Messele T, Mariam HG, et al. Evaluation of the World Health Organization staging system for HIV infection and disease in Ethiopia: association between clinical stages and laboratory markers. AIDS Lond Engl. 1999 Feb 25;13(3):381–9.

15. Klotz SA, Nguyen HC, Van Pham T, Nguyen LT, Ngo DTA, Vu SN. Clinical features of HIV/AIDS patients presenting to an inner city clinic in Ho Chi Minh City, Vietnam. Int J STD AIDS. 2007 Jul;18(7):482–5.

16. Lebelonyane R, Mills LA, Mogorosi C, Ussery F, Marukutira T, Theu J, et al. Advanced HIV disease in the Botswana combination prevention project: prevalence, risk factors, and outcomes. AIDS Lond Engl. 2020 Dec 1;34(15):2223–30.

17. Morpeth SC, Crump JA, Shao HJ, Ramadhani HO, Kisenge PR, Moylan CA, et al. Predicting CD4 lymphocyte count <200 cells/mm(3) in an HIV type 1-infected African population. AIDS Res Hum Retroviruses. 2007 Oct;23(10):1230–6.

18. Nyagaka B, Musyoki SK, Karani L, Nyamache AK. Characteristics and treatment outcomes of HIV infected elderly patients enrolled in Kisii Teaching and Referral Hospital, Kenya. Afr Health Sci. 2020 Dec;20(4):1537–45.

19. Oudenhoven HPW, Meijerink H, Wisaksana R, Oetojo S, Indrati A, van der Ven AJAM, et al. Total lymphocyte count is a good marker for HIV-related mortality and can be used as a tool for starting HIV treatment in a resource-limited setting. Trop Med Int Health TM IH. 2011 Nov;16(11):1372–9.

20. Reda AA, Biadgilign S, Deribew A, Gebre B, Deribe K. Predictors of change in CD4 lymphocyte count and weight among HIV infected patients on anti-retroviral treatment in Ethiopia: a retrospective longitudinal study. PloS One. 2013;8(4):e58595.

21. Sempa JB, Kiragga AN, Castelnuovo B, Kamya MR, Manabe YC. Among Patients with Sustained Viral Suppression in a Resource-Limited Setting, CD4 Gains Are Continuous Although Gender-Based Differences Occur. PLOS ONE [Internet]. 2013 Aug 27 [cited 2024 Dec 12];8(8):e73190. Available from: https://journals.plos.org/plosone/article?id=10.1371/journal.pone.0073190

22. Tassie JM, Marquardt T, Damisoni H, Odhiambo OD, Mulemba M, Szumilin E, et al. Indirect markers to initiate highly active antiretroviral therapy in a rural African setting. AIDS Lond Engl. 2004 May 21;18(8):1226–8.

23. Torpey K, Lartey M, Amenyah R, Addo NA, Obeng-Baah J, Rahman Y, et al. Initiating antiretroviral treatment in a resource-constrained setting: does clinical staging effectively identify patients in need? Int J STD AIDS. 2009 Jun;20(6):395–8.

24. Visser ME, Maartens G, Kossew G, Hussey GD. Plasma vitamin A and zinc levels in HIV-infected adults in Cape Town, South Africa. Br J Nutr. 2003 Apr;89(4):475–82.

25. McGrath N, Kranzer K, Saul J, Crampin AC, Malema S, Kachiwanda L, et al. Estimating the need for antiretroviral treatment and an assessment of a simplified HIV/AIDS case definition in rural Malawi. AIDS [Internet]. 2007 Nov [cited 2025 Jan 24];21(Suppl 6):S105–13. Available from: https://journals.lww.com/00002030-200711006-00014
